# Synthesis, cytotoxicity, anti-inflammatory, anti-metastatic and anti-oxidant activities of novel chalcones incorporating 2-phenoxy-*N*-arylacetamide and thiophene moieties: induction of apoptosis in MCF7 and HEP2 cells

**DOI:** 10.1007/s00210-024-03255-9

**Published:** 2024-07-09

**Authors:** Nada S. Ibrahim, Hager Ahmed Sayed, Marwa Sharaky, Hadeer M. Diab, Ahmed H. M. Elwahy, Ismail A. Abdelhamid

**Affiliations:** 1 Department of Chemistry (Biochemistry Division), Faculty of Science, Cairo University, Giza, 12613 Egypt; 2 Pharmacology unit, Department of Cancer Biology, National Cancer Institute, Cairo University, Cairo, Egypt; 3 Department of Chemistry, Faculty of Science, Cairo University, Giza, 12613 Egypt

**Keywords:** Chalcones, Anti-oxidant, Anti-inflammatory, Wound healing, Intrinsic and extrinsic apoptosis

## Abstract

Eight Novel chalcones were synthesized and their structures were confirmed by different spectral tools. All the prepared compounds were subjected to SRB cytotoxic screening against several cancer cell lines. Compound **5c** exerted the most promising effect against MCF7 and HEP2 cells with IC_50_ values of 9.5 and 12 µg/mL, respectively. Real-time PCR demonstrated the inhibitory effect of compound **5c** on the expression level of *Antigen kiel 67 (KI-67), Survivin, Interleukin-1beta* (*IL-1B*)*, Interleukin-6* (*IL-6*), *Cyclooxygenase-2 (COX-2*) and *Protein kinase B (AKT1)* genes. Flow-cytometric analysis of the cell cycle indicated that compound **5c** stopped the cell cycle at the G0/G1 and G2/M phases in MCF7 and HEP2 treated cells, respectively. ELISA assay showed that Caspase 8, Caspase 9, P53, BAX, and Glutathione (GSH) were extremely activated and Matrix metalloproteinase 2 (MMP2), Matrix metalloproteinase 9 (MMP9), BCL2, Malondialdehyde (MDA), and IL-6 were deactivated in **5c** treated MCF7 and HEP2 cells. Wound healing revealed that chalcone **5c** reduced the ability to close the scrape wound and decreased the number of migrating MCF7 and HEP2 cells compared to the untreated cells after 48 h. Theoretical molecular modeling against P53 cancer mutant Y220C and Bcl2 showed binding energies of -22.8 and -24.2 Kcal/mole, respectively, which confirmed our ELISA results.

**Supplementary Information:**

The online version contains supplementary material available at 10.1007/s00210-024-03255-9.

## Introduction

**Fig. 1:**
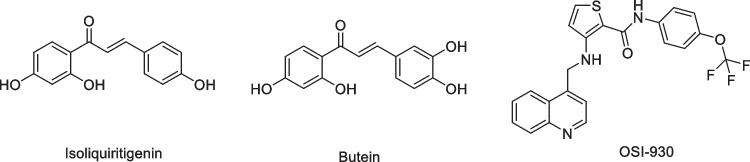
Chemical structures of some naturally occurring chalcones and marketed drug containing thiophene nucleus

**Scheme 1:**
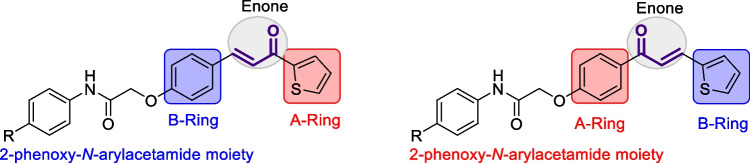
Design strategy of the synthesized compounds

Chalcones are found in conjugated form, with the keto-ethylenic system connecting the two rings (A and B) (Lemes et al. 2020). It is believed that these compounds' biological activity results from the double bond's conjugation with the carbonyl group. Research on chalcones is still in progress because of its many biological properties, which include anti-oxidant, (Bandgar et al. 2009; Shenvi et al. 2013) anti-bacterial, (Asiri and Khan 2011; Mohamed et al. 2012) antiviral, (Onyilagha et al. 1997) anti-platelet, (Lin et al. 2001) anti-cancer, (Sashidhara et al. 2010; Shenvi et al. 2013) anti-malarial, (Li et al. 1995) analgesic, (Heidari et al. 2009) and anti-inflammatory properties (Hsieh et al. 2000; Bekhit and Abdel-Aziem 2004). Moreover, thiophenes are sulfur-containing heterocycles that play an essential role in medicinal chemistry due to their wide range of biological applications, including anti-cancer, (Duddukuri et al. 2018) anti-microbial, (Kheder et al. 2008; Bondock et al. 2010) anti-inflammatory, (Helal et al. 2015) activities. Figure 1 shows some anti-cancer natural chalcones and anticancer agents that contain thiophene nuclei. Furthermore, it was noted that compounds having an acetamide linker or its substitutes as essential structures have gained substantial interest due to possible medicinal uses, including anti-oxidant (Ölgen et al. 2013), anti-cancer (Bhavsar et al. 2011; Khazir et al. 2020), analgesic (Yusov et al. 2019; Mikhailovskii et al. 2020), anti-microbial (Gull et al. 2016; Mikhailovskii et al. 2020; Yele et al. 2021), anti-inflammatory (Yusov et al. 2019), anti-urease (Gull et al. 2016), anti-tuberculosis (Borsoi et al. 2020), anti-convulsant (Severina et al. 2020), anti-COVID-19 (Mary et al. 2021), and anti-tubercular agents (Ang et al. 2012). Some acetamide derivatives have been shown to have analgesic or sedative characteristics, such as paracetamol (Rani et al. 2014), which is one of the most commonly used antipyretic and sedative drugs. In addition, AdipoRon, a phenoxyacetamide medication, has attracted great interest as a possible therapy for obesity, heart disease, diabetes, and non-alcoholic fatty liver (Akimoto et al. 2018). In light of these results and our ongoing research interest in the synthesis of bioactive heterocycles (A. Ibrahim et al. 1994; Barsoum et al. 1998; Elwahy and Abbas 2000; Elwahy et al. 2002; Ibrahim et al. 2004; Al-Awadi et al. 2007; Darwish et al. 2010; Mekky and Elwahy 2014; Ghozlan et al. 2015; Sayed et al. 2016; Ibrahim et al. 2018; Mohamed et al. 2018; Sroor et al. 2019; Fathi et al. 2021; Helmy et al. 2022; WalyEldeen et al. 2023; Abdelwahab et al. 2024; Abdullah et al. 2024; Elwahy et al. 2024a; Ragheb et al. 2024a; Saleh et al. 2024; Salem et al. 2024a, b; Ragheb et al. 2024b; Elwahy et al. 2024b) we were motivated to synthesize novel chalcones incorporating 2-phenoxy-*N*-arylacetamide and thiophene moieties and evaluate their *in vitro* anti-cancer efficacy against different human cancer cell lines (Scheme 1).

## Results and Discussion

**Scheme 2:**
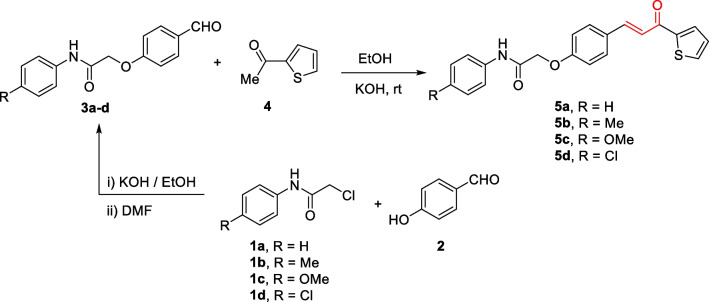
Synthesis of 2-(4-(3-oxo-3-(thiophen-2-yl)prop-1-en-1-yl)phenoxy)-*N*-arylacetamides **5a–d**

**Scheme 3:**
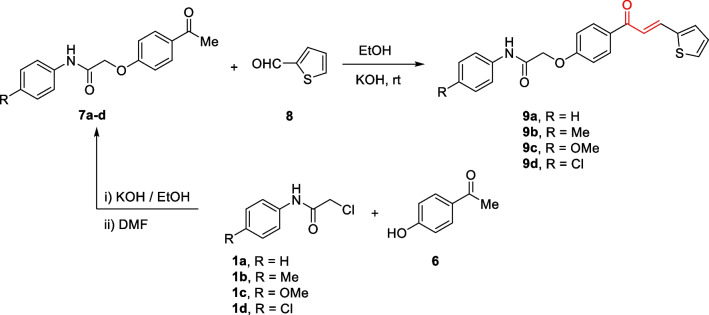
Synthesis of *N*-aryl-2-(4-(3-(thiophen-2-yl)acryloyl)phenoxy)acetamides** 9a–d**

The 2-(4-formylphenoxy)-*N*-arylacetamide precursors **3a–d** (Omar et al. 2021; Abdelwahab et al. 2023) were produced by the alkylation reaction involving 4-hydroxybenzaldehyde **2** with the corresponding 2-chloro-*N*-arylacetamide **1** in the presence of KOH, as indicated in Scheme 2. The formation of chalcone incorporating 2-phenoxy-*N*-arylacetamide, (2-(4-(3-oxo-3-(thiophen-2-yl)prop-1-en-1-yl)phenoxy)-*N*-arylacetamide **5a–d** was due to of the Claisen-Schmidt condensation reaction of precursors **3a–d** with the mole equivalent of 1-(thiophen-2-yl)ethan-1-one 4 in ethanol in the presence of KOH at reflux. In the formed chalcones **5a–d**, thiophene represents A-ring, while 2-phenoxy-*N*-arylacetamide represents B-ring (as shown in scheme 3). The constitutions of the resulting products were verified based on spectral data.

Motivated by the results obtained in scheme 2, we prepared the isomeric *N*-aryl-2-(4-(3-(thiophen-2-yl)acryloyl)phenoxy)acetamides **9a–d**, in which thiophene represents B-ring, while 2-phenoxy-*N*-arylacetamide represents A-ring. The 2-(4-acetylphenoxy)-*N*-arylacetamide precursors **7a–d**, were prepared *via* the alkylation reaction of 4-hydroxyl acetophenone **6** with the corresponding 2-chloro-*N*-arylacetamides **1** in the presence of KOH as shown in scheme 3. Claisen-Schmidt condensation reaction of 2-(4-acetylphenoxy)-*N*-arylacetamide precursors **7a–d** (Abdullah 2023) with the mole equivalent of thiophene-2-carbaldehyde 8 in ethanol in the presence of KOH at reflux resulted in the formation of **9a–d** (as shown in Scheme 3).

A spectrochemical study confirmed the chemical structures of the new compounds **5a–c** and **9a–c**. The IR spectra of compound **9b**, as a sample example, revealed the existence of the carbonyl band at 1691 and 1666 cm^-1^. The mass spectra for **9b** also revealed the right molecular ion peak at m/z 377. Furthermore, in 9b's ^1^H NMR spectra, two singlet signals at 2.27 ppm and 4.83 ppm, were assigned to tolyl CH_3_ and OCH_2_, respectively. The structure of the compound was assigned as *trans* configuration as it revealed to doublets at 7.57 and 7.90 ppm with coupling constant, *J* 20 Hz. At 10.03 ppm, amide-NH appeared as a broad signal. The chemical shifts of all other protons and carbons were exactly as expected.

### Cytotoxic assay

#### Primary screening

All the prepared compounds were screened at a concentration of 100 µg/mL against six different human cell lines: human laryngeal carcinoma (HEP2), human colorectal carcinoma (HCT_116_), human breast carcinoma (MCF7), human Lung carcinoma (A549), human liver carcinoma (HEPG2), and normal African Green monkey kidney cell line (VERO). Doxorubicin was used as a positive control for comparison purposes. Table 1 showed that compounds **5c** and **9a** exerted the most promising activity against MCF7 and HEP2 cells which showed % inhibition of more than 75%. Also, compounds **5c** and **9a** showed an inhibition of 58% against the VERO normal cell line. The remaining compounds did not show cytotoxic activity against all the studied cancer cell lines which inhibited lower than 50% of cancer cells (Table 1). So, secondary screening was performed on the most effective compounds (**5c** and **9a**) to determine their IC_50_ values.

**Table 1:** % Inhibition in different cancer cell lines after the treatment with a single dose (100µg/mL) of the prepared chalcone derivatives

			**%**Inhibition at100 µg/mL			
Compound	HEP2	HCT116	MCF7	A549	HEPG2	VERO
**5a**	4.53±0.08	5.18±1.6	33.70±1.5	22±1.4	30±1.2	55±3
**5b**	7.20±1.4	5.92±1.2	0.52±2.1	10.2±2	25±1.9	60±1
**5c**	79.93±1.1	41.91±1.8	87.30±1.7	65 ±2.1	67±1.6	58±1.5
**5d**	0.36±0.05	0.85±1.1	40.09±0.07	44.1±1.3	45.21±1.7	80±2
**9a**	78.70±0.5	44.66±1.0	81.53±0.09	52±2.7	64±1.5	58±1.4
**9b**	17.89±1.3	0.92±1.7	18.60±0.05	33±3.1	26±1.7	15±2.4
**9c**	7.20±1.7	16.29±1.9	1.81±1.3	IA	IA	IA
**9d**	17.89±2.3	6.66±3.3	2.28±4.1	IA	IA	10±1
Doxorubicin	83.25±1.1	71.55±0.9	86.56±1.6	77±1.5	81±2	45±2

IA means inactive

#### Secondary screening

**Fig. 2:**
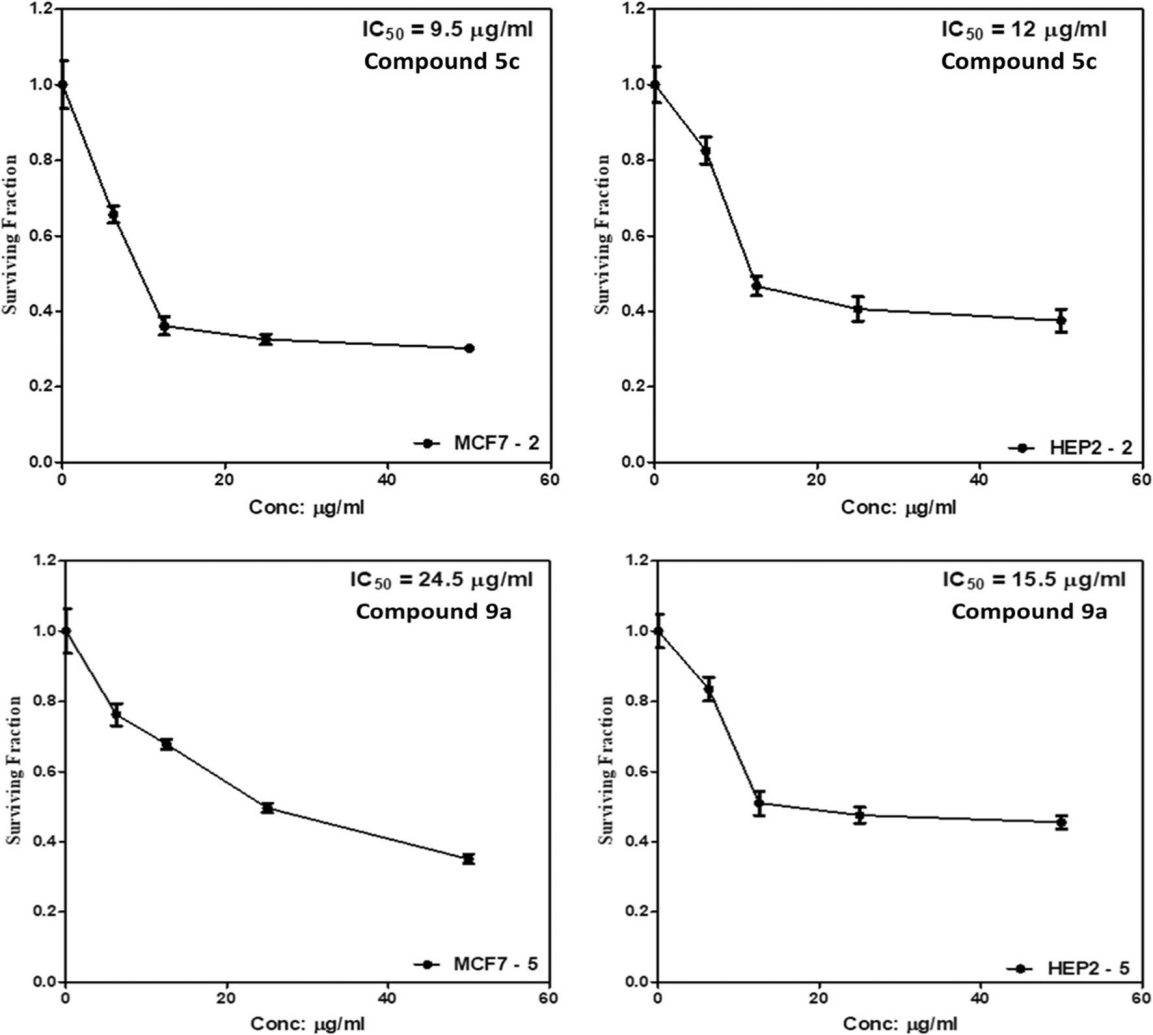
Impact of **5c** and **9a** on the viability of MCF7 and HEP2 cells using SRB assay. GraphPad Prism, version 5, was used to create graphs and analyze the data. The results are expressed as the mean ± SD of 3 separate experiments performed in 5 replicates

As shown in Fig. 2 and Table 2, *E*)-*N*-(4-Methoxyphenyl)-2-(4-(3-oxo-3-(thiophen-2-yl)prop-1-en-1-yl)phenoxy)acetamide **5c** in which the *N*-arylacetamide has electron-donating methoxy group on B-ring **5c** exerted better activity than compound **9a** against MCF7 and HEP2 cells. In terms of IC_50_ values, compound **5c** displayed 12 and 9.5 µg/mL against HEP2 and MCF7 cells, which were very comparable to that of doxorubicin (11 and 5.5 µg/mL), respectively. In addition, compound **5c** was better than two reported chalcone derivatives ((*E*)-1-(4-nitrophenyl)-3-(4-(hexyloxy)phenyl) prop-2-en-1-one and (*E)*-1-(4-cyanophenyl)-3-(4-(hexyloxy)phenyl) prop-2-en-1-one) as they exerted cytotoxic effect against MCF7 with IC_50_ values of 14.75 and 13.75 µg/mL, respectively (Khairul et al. 2020). While, compound **9a** exerted 15.5 and 24.5 µg/mL against HEP2 and MCF7, respectively. Therefore, subsequent molecular studies were conducted on compound **5c** against MCF7 and HEP2 cells.

**Table 2:** IC_50_ values (µg/mL) of compounds **5c** and **9a** against HEP2 and MCF7 cells

IC_50_ (µg/mL)		
Compound	HEP2	MCF7
**5c**	**12**	**9.5**
**9a**	**15.5**	**24.5**
Doxorubicin	**11**	**5.5**

### RT-PCR

The expression level of the following six genes (*Ki-67, Survivin,* Interleukin-1 beta (*IL-1B*)*,* Interleukin-6 (*IL-6*)*,* Cyclooxygenase-2 (*COX-2*) and protein kinase B (*AKT1*)) was determined in MCF7 and HEP2 treated with the IC_50_ of compound **5c**. The untreated cells were used as a negative control for comparison purposes. Data illustrated in Table 3 showed that chalcone **5c** had a significant inhibitory effect on the expression of these genes in MCF7 and HEP2-treated cells relative to their untreated control cells. *Ki-67* is a popular proliferation marker for human tumor cells. It presents in all active phases of the cell cycle and its cellular distribution substantially alters as the cell cycle advances (Luo et al. 2021) It was down-regulated by **5c** in MCF7 and HEP2 cells with fold changes of 0.352±0.0172 and 0.648±0.015, respectively, relative to their controls. *Survivin* is an inhibitor of apoptotic protein (IAP) family member, which is essential for cell division. The lowering of the expression of *survivin* inhibited tumor development, triggered apoptosis, and made tumor cells more susceptible to radiation and chemotherapy (Albadari and Li 2023). It was found that **5c** decreased the expression level of *survivin* in MCF7 and HEP2 cells with values (0.632±0.0618 and 0.489±0.07), respectively, relative to their controls. IL-1B is a pleiotropic mediator of inflammation, which is produced in response to a variety of stressors (Aarreberg et al. 2019). Herein, it was down-regulated in **5c** treated MCF7 and HEP2 cells with values (0.4008±0.116 and 0.461±0.069) respectively, relative to their controls. IL-6 is a pro-inflammatory cytokine that plays a significant role in the proliferation and differentiation of human cells. It promotes the production of numerous proteins involved in acute inflammation (Uciechowski and Dempke 2020). It was found that **5c** lowered the expression level of IL-6 in MCF7 and HEP2 cell lines with values (0.814±0.079 and 0.464±0.08), respectively relative to their untreated control cells. COX-2 is a vital physiological enzyme that is essential for many biological processes, particularly in the mechanisms involved in pain and inflammation. The overexpression of COX-2 is found to be related to inflammatory processes and cancer (Sharma et al. 2019). It was showed that **5c** down-regulated COX-2 in MCF7 and HEP2 treated cells with approximately similar values (0.502±0.073 and 0.547±0.0321), respectively, relative to their controls. AKT1 has a critical role in influencing several pathways, including preventing apoptosis, promoting cell proliferation, and altering cellular metabolism (Ghafouri-Fard et al. 2022). Chalcone **5c** greatly decreased the expression level of AKT1 in MCF7 and HEP2 treated cells with fold changes (0.402±0.068 and 0.381±0.011), respectively, relative to their controls. So, the inhibitory effect of chalcone **5c** on the expression level of KI-67, Survivin, IL-1B, IL-6, COX-2, and AKT1 promoted the apoptotic death and blocked the inflammation in MCF7 and HEP2 cells.

**Table 3:** RT-PCR data demonstrated the expression folding ±SD of (*KI67, Survivin, IL-1B, IL-6, COX2,* and *AKT1*) genes in compound **5c** treated MCF7 and HEP2 cells

Sample	Untreated MCF7	MCF7/ 5c	Untreated HEP2	HEP2/ 5c
KI67	1±0	0.352±0.0172	1±0	0.648±0.015
Survivin	1±0	0.632±0.0618	1±0	0.489±0.07
IL-1B	1±0	0.4008±0.116	1±0	0.461±0.069
IL-6	1±0	0.814±0.079	1±0	0.464±0.08
COX2	1±0	0.502±0.073	1±0	0.547±0.0321
AKT1	1±0	0.402±0.068	1±0	0.381±0.011

### ELISA assay

The concentrations of matrix metalloproteinase-2 (MMP2), matrix metalloproteinase-9 (MMP9), Bcl2, BAX, P53, Malondialdehyde (MDA), Glutathione (GSH), caspase 8, caspase 9 and IL-6 were measured quantitatively in **5c** treated MCF7 and HEP2 cells using ELISA (Enzyme-Linked Immunosorbent Assay) (Table 4). Gelatinases are a collective word for MMP-2 and MMP-9, where MMP-2 is gelatinase-A and MMP-9 is gelatinase-B. Solid tumor invasion, metastasis, and angiogenesis have long been linked to gelatinases (Das et al. 2021). Our study showed that chalcone **5c** decreased the activity of MMP2 and MMP9 in MCF7 and HEP2 cells with values (185.125 and 142.5 pg/mL), respectively, for MMP2 and (271.6 and 236.5 ng/mL), respectively for MMP9 relative to their controls. Bcl2 is a crucial protein that targets apoptosis inhibition. It is regarded as a predictive biomarker or therapeutic target in the diagnosis of cancer due to its broad expression in a variety of cancers (Porter et al. 2009). It was found that Bcl2 was deactivated in MCF7 and HEP2 cells treated with chalcone **5c** with values (2089.96 and 1809.34 pg/mL), respectively, relative to their controls. The pro-apoptotic protein BAX permeabilizes the mitochondrial outer membrane by changing from a cytosolic monomer to a hazardous oligomer triggering the apoptosis process (Fulda and Debatin 2006). Herein, **5c** increased the concentration of BAX in MCF7 and HEP2 cells with values (1099.36 and 1342.8 pg/mL), respectively, relative to their controls. P53 transcription factor performs an essential tumor suppressor role by coordinating a wide range of physiological responses, including DNA repair, cell cycle arrest, cellular senescence, cell death, cell differentiation, and metabolism (Liebl and Hofmann 2021). It was activated in MCF7 and HEP2-treated cells with values (4.28 and 3.109 ng/mL), respectively, compared to their controls (3.44 and 2.06 ng/mL**)**. MDA is the main biomarker for determining lipid peroxidation. MDA is a final product of polyunsaturated fatty acids (PUFAs) peroxidation, either through healthy or pathological enzyme- or non-enzyme-catalyzed processes (Chakravarty and Rizvi 2011). Our study demonstrated that **5c** decreased the concentration of MDA in MCF7 and HEP2 cells with values (8.79 and 11.988 nM), respectively, relative to their controls. GSH is an anti-oxidant that performs a variety of physiological tasks, such as scavenging free radicals, fighting oxidation, and getting rid of electrophiles (Chakravarty and Rizvi 2011). When the ROS/GSH equilibrium is upset, bio-macromolecules are negatively oxidized and chemically modified, which ultimately causes cell cycle arrest, proliferation inhibition, and even cell death. A direct rise in ROS may lead to an imbalanced ROS/GSH ratio. GSH was extremely activated in MCF7 and HEP2 cells treated with **5c** with values (0.939 and 0.821 ng/mL), respectively, compared to their controls. The extrinsic apoptotic process is often triggered by caspase-8, a cysteine-aspartate-specific protease when cell surface death receptors (DRs) like FAS, TRAIL-R, and TNF-R are activated (Mandal et al. 2020). In addition to its activities in death receptor-mediated apoptosis, Caspase-8 also inhibits a different type of programmed cell death known as necroptosis, which is an inflammatory cell death (Mandal et al. 2020). It was found that **5c** strongly activated caspase-8 in MCF7 and HEP2 cell lines with values (2.13 and 2.27 ng/mL), respectively, relative to their untreated control cells. Caspase-9 is a crucial component of the intrinsic or mitochondrial apoptotic pathway, which is activated by a variety of stimuli such as chemotherapy, stress medications, and radiation (Li et al. 2017). Herein, caspase-9 was up-regulated in MCF7 and HEP2 cell lines treated with **5c** with values (28.03 and 27.54 ng/mL), respectively, relative to their controls. Also, it was found that IL-6 was deactivated in MCF7 and HEP2-treated cells with values (74.68 and 54.27 pg/mL), respectively, as compared to their controls. This result supported the downregulating effect of **5c** on the expression level of *IL-6* as mentioned in the RT-PCR section. So, Caspase 8, Caspase 9, P53, BAX, and GSH were extremely activated and MMP2, MMP9, BCL2, MDA, and IL-6 were deactivated in **5c** treated MCF7 and HEP2 cells. From the above results, we could suggest that compound **5c** triggered both intrinsic and extrinsic pathways of apoptosis in MCF7 and HEP2 cells. Also, it could inhibit invasion, metastasis, and inflammation and had anti-oxidant activity in treated MCF7 and HEP2 cells.

**Table 4:** Concentrations ±SD of (MMP2, MMP9, Bcl2, BAX, P53, MDA, GSH, Caspase 8, Caspase 9 and IL-6) in **5c** treated MCF7 and HEP2 cells

Sample	Untreated MCF7	MCF7/**5c**	Untreated HEP2	HEP2/**5c**
MMP2 (pg/mL)	466.90±22.55	185.125±30.07	461.58±45.11	142.5±15.03
MMP9 (ng/mL)	588.28±24.87	271.6±58.04	564.8±41.45	236.5±24.87
Bcl2 (pg/mL)	2479.8±17.08	2089.96±17.97	2299.4±170.35	1809.34±27.45
BAX (pg/mL)	946.30±12.7	1099.36±12.8	1261.8±7.38	1342.8±12.7
P53 (ng/mL)	3.44±0.25	4.28±0.188	2.06±0.425	3.109±0.089
MDA (nM)	15.79±0.178	8.79±0.309	17.44±0.309	11.988±0.178
GSH (ng/mL)	0.737±0.009	0.939±0.022	0.668±0.010	0.821±0.009
Caspase8 (ng/mL)	0.162±0.0203	2.13±0.268	0.187±0.025	2.27±0.142
Caspase9 (ng/mL)	3.66±0.49	28.03±0.49	3.70±0.18	27.54±0.68
IL-6 (pg/mL)	100.47±0.439	74.68±0.979	85.24±1.16	54.27±5.509

### Flow cytometric analysis of cell cycle

#### Compound 5c induces cell cycle arrest at the G0-G1 phase in MCF-7 cells

**Fig. 3:**
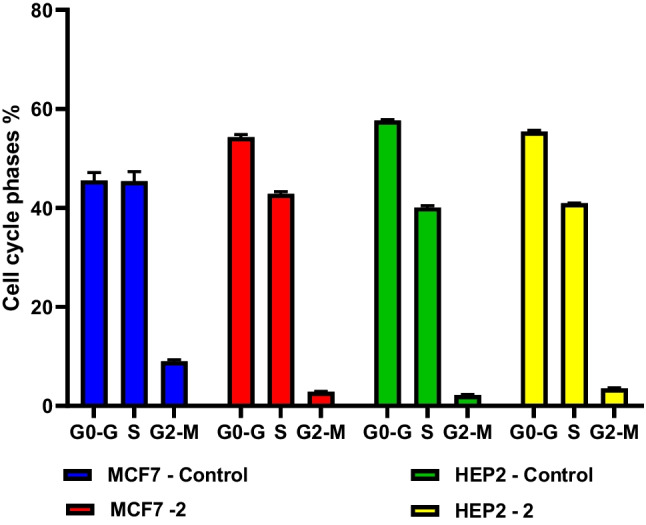
Distribution of cells in different phases of the cell cycle after the treatment of MCF7 and HEP2 cells with the IC_50_ value of **5c** relative to the untreated control cells

The percentage of cells in the G0-G1 phase increased from 45.5% in the untreated control MCF7 cells to 54.3% in the **5c**-treated cells, as shown in Table 5 and Fig. 3. In the G2-M and S phases, fewer **5c**-treated cells were seen at 2.8% and 42.84%, respectively, compared to the untreated control with 8.9% and 45.4%, and this was compatible with the results obtained by the literature (Gao et al. 2020) where Xanthohumol caused cell cycle arrest at G0-G1phase.

**Table 5:** Percentage of DNA in different phases of the cell cycle for **5c** treated MCF7 and HEP2 cells relative to their untreated control cells, Values are the means±SD

Sample	G0-G1	S	G2-M
Untreated MCF7 (control)	45.585±1.548	45.405±1.97283	8.95±0.38
MCF7/ **5c**	54.3±0.5374	42.845±0.45962	2.855±0.07778
Untreated HEP2 (control)	57.67±0.21213	40.095±0.36062	2.205±0.10607
HEP2/ **5c**	55.475±0.233	40.96±0.08485	3.565±0.14849

#### Compound 5c induces cell cycle arrest at the G2/M phase in HEP2 cells

According to Table 5 and Fig. 3, the percentage of cells in the G2/M phase increased from 2.2% to 3.56% in the **5c**-treated cells compared to the untreated control HEP2 cells, and the percentage of cells in the S phase increased slightly from 40.09% to 40.96%. In the G0-G1 stage, the percentage of 5c-treated cells declined to 55.4% as opposed to 57.6% of untreated control cells. It was found in a previous work that lutein-induced G2/M phase arrest in A549 and PC-9 cells (Di et al. 2019).

#### Compound 5c inhibits MCF7 and HEP2 cells migration

**Fig. 4:**
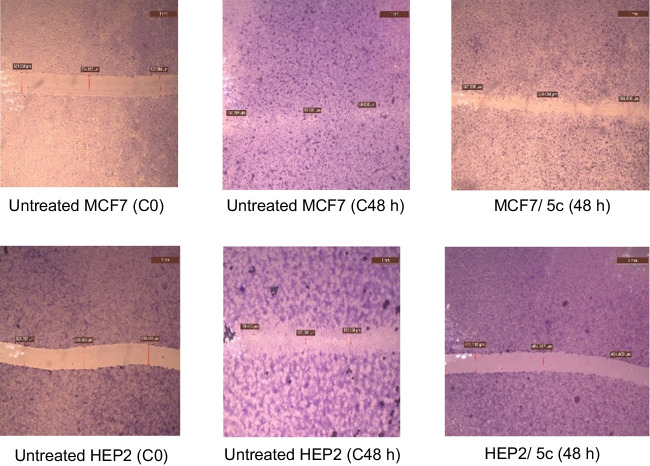
Evaluation of migration capacity using a wound‑healing assay following compound **5c** treatment for 48 h in MCF7 and HEP2 cells

To examine the effect of chalcone **5c** on the migration properties of the MCF7 and HEP2 cells, the wound-healing scratch assay was used. The untreated MCF7 and HEP2 control (C) cells generally displayed wound recovery within 48 h and migration of cells to the wound (Fig. 4 and Table 6). Chalcone **5c** at its IC_50_ reduced the ability to close the scrape wound and decreased the number of migrating MCF7 and HEP2 cells compared to the untreated cells (Fig. 4 and Table 6). The scratch gap percentage in **5c** treated MCF7 was 344.684±41.224 relative to the control cells (160.647±61.276) after 48 h of treatment. The scratch gap in HEP2 treated with **5c** was 567.281±112.789 compared to the untreated control HEP2 cells (211.604±79.883) after 48 h. This result supported the lowering effect of **5c** on the concentration of MMP-2 and MMP-9 in MCF7 and HEP2 cells as shown in the ELISA section. Our result coincided with the result of Luo et al (Luo et al. 2021), where two ligustrazine-chalcone hybrids (compounds **6c** and **6f**, therein) inhibited significantly the migration of MDA-MB-231 and MCF-7 cells in a concentration-dependent manner.

**Table 6:** Migration ability determination in **5c** treated MCF7 and HEP2 cells after 48 h. The values are the mean±SD

% Scratch gap		
Sample	MCF7	HEP2
Untreated (C0)	518.531±12.437	650.844±127.510
Untreated (C48 h)	160.647±61.276	211.604±79.883
**5c** (48 h)	344.684±41.224	567.281±112.789

### Molecular docking

**Fig. 5:**
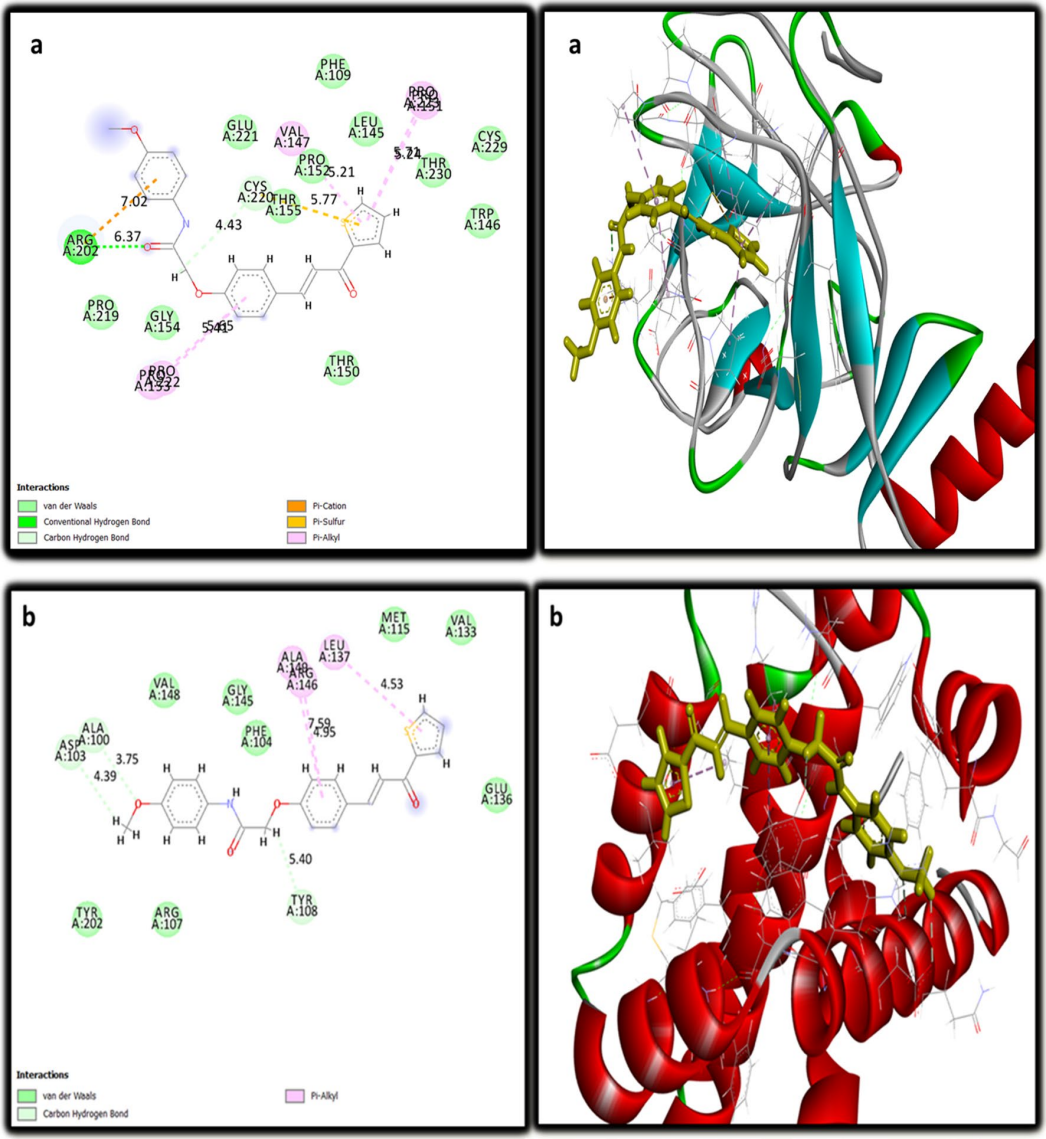
Two-dimensional and three-dimensional mode of interaction of compound **5c** with the active site of **a**) P53 cancer mutant Y220C; **b**) Bcl2

**Fig. 6:**
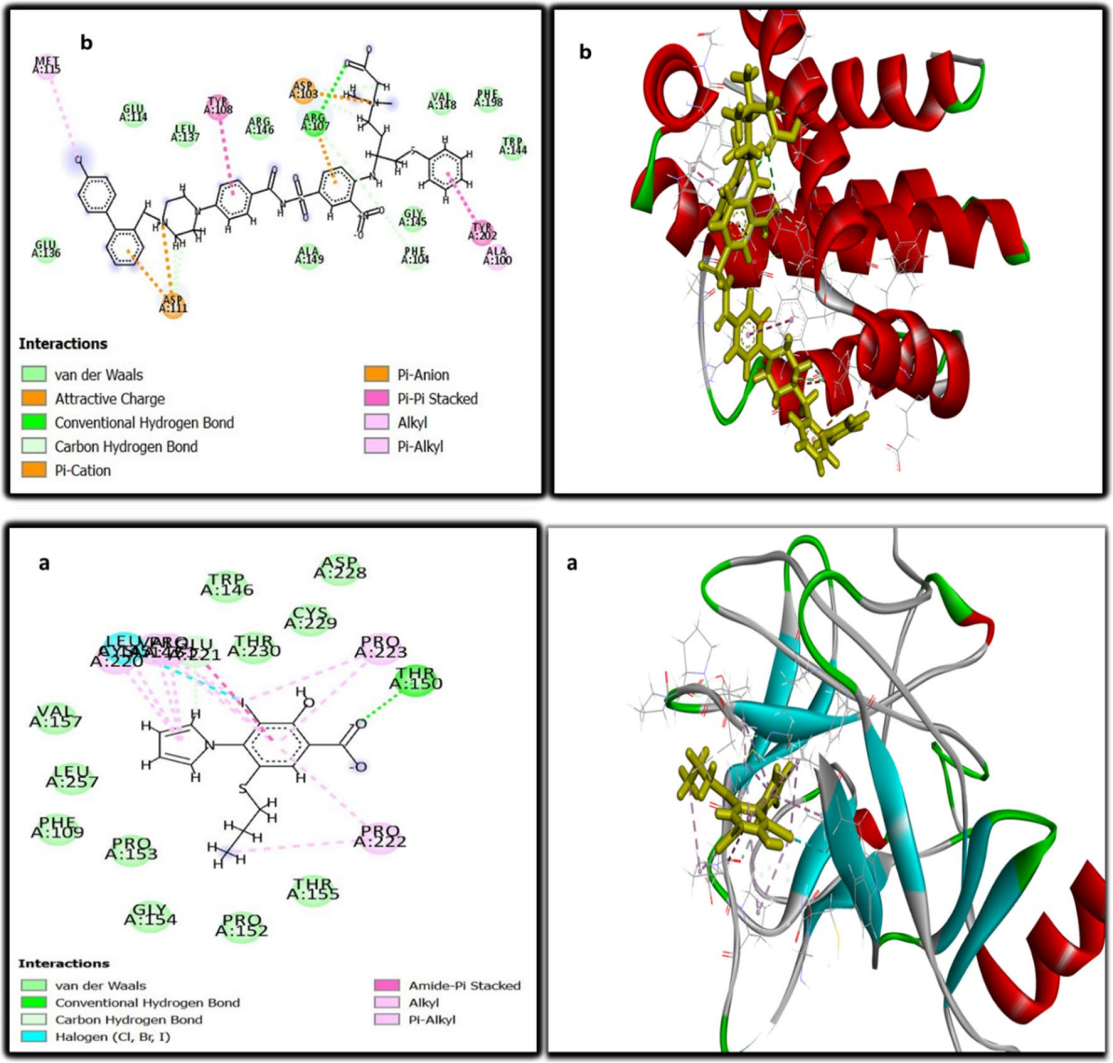
Two-dimensional and three-dimensional mode of interaction of the standard ligands with the active site of **a**) P53 cancer mutant Y220C; **b**) Bcl2

The molecular docking study was done on the promising compound **5c** against P53 cancer mutant Y220C and Bcl2 proteins. As shown in Table 7, the values of the binding energy of the studied compound **5c** were -22.8 and -24.2 Kcal/mole, respectively, which were more negative and better than that of the standard co-crystallized ligand (-15.8 and -21.83 Kcal/mole), respectively. The root mean squared deviations (RMSDs) were 0.7 and 2.5 for P53 cancer mutant Y220C and Bcl2, respectively. Compound **5c** interacted with the active site of P53 cancer mutant Y220C *via* 9 interactions (Fig. 5a). These interactions included one carbon-hydrogen bond between the hydrogen of the methylene moiety and CYS:220 with a bond distance of 4.43 A°; a conventional hydrogen bond between the oxygen of the carbonyl group and ARG:202 with a bond distance of 6.37 A°; one pi-cation electrostatic interaction between the benzene ring and ARG:202 with bond distance 7.02 A°; one pi-sulfur electrostatic interaction between the thiophene ring and CYS:220 with bond distance 5.77 A°; and five pi-alkyl hydrophobic interactions with PRO:222, PRO:153, PRO:223, PRO:151 and VAL:147 residues. Compound **5c** interacted with the active site of Bcl2 through six interactions (Fig. 5b). These interactions included three carbon-hydrogen bonds with ALA: 100, ASP: 103, and TYR: 108 with bond distances 3.75, 4.39, and 5.40 A°, respectively; and 3 pi- alkyl hydrophobic interactions with LEU: 137, ALA: 149, and ARG: 146 with bond distances 4.53, 7.59, and 4.95 A°, respectively. Figure 6a showed the interaction of 3-iodanyl-2-oxidanyl-5-propylsulfanyl-4-pyrrol-1-yl-benzoicacid (standard ligand) with P53 cancer mutant Y220C which revealed one conventional hydrogen bond with THR: 150 with bond distance 2.04 A°; carbon-hydrogen bond with GLU: 221; and twelve hydrophobic interactions including (amide pi-stacked, alkyl, pi-alkyl and halogen). Figure 6b demonstrated eleven interactions between Bcl2 and its co-crystallized ligand which included four hydrogen bonds; four electrostatic; and three hydrophobic interactions. So, the above results indicated that compound **5c** had an activating effect on P53 mutant Y220C and an inhibitory effect against Bcl2 anti-apoptotic protein, and this assumption coincided with our results in the above ELISA assay section.

**Table 7:** Values of the binding energy (Kcal/mole) of compound **5c** with the active domains of P53 cancer mutant Y220C and Bcl2 compared to the values of standard ligand

Binding energy (S) (Kcal/mole)		
Compound	P53 cancer mutant Y220C	Bcl2
**5c**	-22.8	-24.2
Standard ligand	-15.8	-21.83

## Experimental

### Chemistry

“Melting points were measured with a Stuart melting point apparatus and were uncorrected. The IR spectra were recorded using an FTIR Bruker–vector 22 spectrophotometer as KBr pellets. The ^1^H and ^13^C NMR spectra were recorded in DMSO as a solvent on a Brucker spectrometer (400 MHz) using TMS as an internal standard. Chemical shifts are reported as *δ* values in ppm. Mass spectra were recorded with a Shimadzu GCMS–QP–1000 EX mass spectrometer in an EI (70 eV) model. The elemental analyses were performed at the Microanalytical Center, Cairo University.

#### General procedures for the synthesis of chalcones 5a-d and 9a-d

A solution consisting of aldehydes (**3a–d** or **8**) or acetyl derivatives (**4** or **7a–d**) (1 mmol) had been dissolved in ethanol (20 mL). The solution of potassium hydroxide (20%, 5 ml) was then added to this mixture at 0-5 °C. The reaction mixture was stirred regularly for 5 hours at room temperature and then transferred over HCl-containing ice. The resulting yellow solid was filtered, rinsed with water, and dried. The crude product was crystallized using EtOH-Dioxane to produce yellow crystals of chalcones **5a–d** and **9a–d**.

#### 2-(4-(3-Oxo-3-(thiophen-2-yl)prop-1-en-1-yl)phenoxy)-*N*-phenylacetamide (5a)


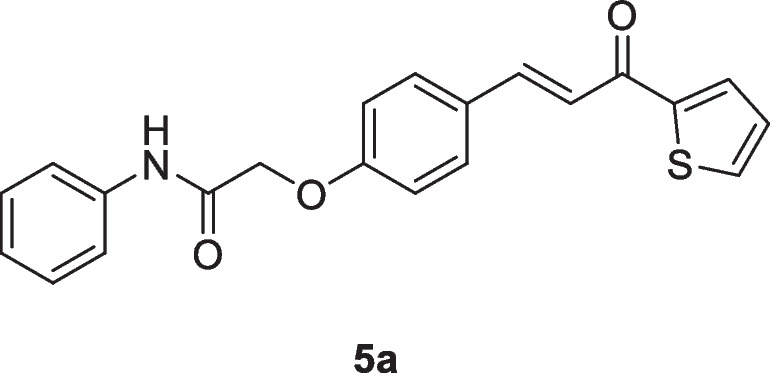


Yellow crystals (85%); mp 194-196 °C; IR (KBr): ν 3366 (NH), 1694 (C=O ketone), 1660 (C=O amide) cm^-1^; ^1^H NMR (400 MHz, DMSO-*d*_*6*_*)*: δ 4.80 (s, 2H, C*H*_2_), 7.09 – 7.11 (m, 3H, Ar-*H* + vinyl-*H* + thiophene-H), 7.31-7.36 (m, 3H, Ar-*H* + vinyl-*H*), 7.64 (d, 2H, Ar-H, *J* = 8Hz), 7.73 (d, 2H, Ar-H, *J* = 12Hz), 7.87 (d, 2H, Ar-H, *J* = 12Hz), 8.04 (m, 1H, thiophene-H), 8.30 (m, 1H, thiophene-H),10.14 (s, 1H, N*H*) ppm; ^13^C NMR (101 MHz, DMSO-*d*_*6*_*)*: δ 67.6 (-OCH_2_CO-), 115.6, 120.1, 120.2, 124.2, 128.2, 129.2, 129.3, 131.2, 133.8, 135.7, 138.8, 143.4, 146.2, 160.4 (ArC-O-CH_2_), 166.6 (-NHCO), 182.0 (-CO) ppm; MS (EI, 70 eV): *m/z* 363 [M]^+^; Anal. Calcd for C_21_H_17_NO_3_S: C, 69.40; H, 4.72; N, 3.85%. Found: C, 69.26; H, 4.63; N, 3.77%.

#### 2-(4-(3-Oxo-3-(thiophen-2-yl)prop-1-en-1-yl)phenoxy)-*N*-(p-tolyl)acetamide (5b)


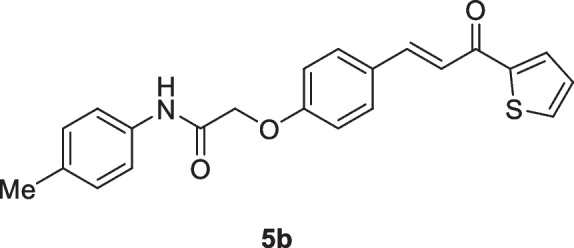


Yellow crystals (84%); mp 202-204 °C; IR (KBr): ν 3362 (NH), 1697 (C=O ketone), 1661 (C=O amide) cm^-1^; ^1^H NMR (400 MHz, DMSO-*d*_*6*_*)*: δ 2.23 (s, 3H, C*H*_3_), 4.83 (s, 2H, C*H*_2_), 7.13 – 7.21 (m, 5H, Ar-*H* + thiophene-H), 7.53 (d, 2H, Ar-H, *J* = 8Hz), 7.56 (d, 1H, vinyl-*H*, *J* = 20Hz), 7.69 (d, 1H, thiophene-H, *J* = 4Hz), 7.77 (d, 1H, thiophene-H, *J* = 4Hz), 7.87 (d, 1H, vinyl-*H*, *J* = 20Hz), 8.12 (d, 2H, Ar-H, *J* = 8Hz), 10.11 (s, 1H, N*H*) ppm; MS (EI, 70 eV): *m/z* 377 [M]^+^; Anal. Calcd for C_22_H_19_NO_3_S: C, 70.01; H, 5.07; N, 3.71%. Found: C, 69.91; H, 5.02; N, 3.60%.

#### *N*-(4-Methoxyphenyl)-2-(4-(3-oxo-3-(thiophen-2-yl)prop-1-en-1-yl)phenoxy)acetamide. (5c)


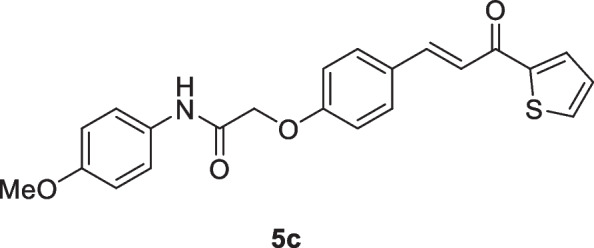


Green crystals (82%); mp 208-210 °C; IR (KBr): ν 3345 (NH), 1692 (C=O ketone), 1659 (C=O amide) cm^-1^; ^1^H NMR (400 MHz, DMSO-*d*_*6*_*)*: δ 3.73 (s, 3H, OC*H*_3_), 4.77 (s, 2H, C*H*_2_), 6.90 – 6.95 (m, 3H, Ar-*H* + vinyl-*H* ), 7.09-7.13 (m, 2H, vinyl-*H* + thiophene-H), 7.54 (d, 2H, Ar-H, *J* = 12Hz), 7.73 (d, 2H, Ar-H, *J* = 12Hz), 7.87 (d, 2H, Ar-H, *J* = 12Hz), 8.04 (d, 1H, thiophene-H, *J* = 4Hz), 8.30 (d, 1H, thiophene-H, *J* = 4Hz), 10.0 (s, 1H, N*H*) ppm; MS (EI, 70 eV): m/z 393 [M]^+^; Anal. Calcd for C_22_H_19_NO_4_S: C, 67.16; H, 4.87; N, 3.56%. Found: C, 67.08; H, 4.74; N, 3.49%.

#### *N*-(4-Chlorophenyl)-2-(4-(3-oxo-3-(thiophen-2-yl)prop-1-en-1-yl)phenoxy)acetamide. (5d)


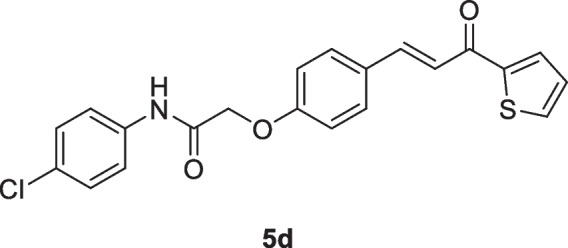


Yellow crystals (78%); mp 198-200 °C; IR (KBr): ν 3337 (NH), 1695 (C=O ketone), 1655 (C=O amide) cm^-1^; ^1^H NMR (400 MHz, DMSO-*d*_*6*_*)*: δ 4.80 (s, 2H, C*H*_2_), ^1^H NMR (400 MHz, DMSO-*d*_*6*_*)*: δ 4.83 (s, 2H, C*H*_2_), 7.07 (d, 2H, Ar-*H*, *J* = 12Hz), 7.29-7.32 (m, 1H, thiophene-H), 7.40 (d, 2H, Ar-H, *J* = 12Hz), 7.66-7.73 (m, 4H, Ar-H + 2 vinyl-*H*), 7.85 (d, 2H, Ar-H, *J* = 12Hz), 8.04 (d, 1H, thiophene-H, *J* = 4Hz), 8.28 (d, 1H, thiophene-H, *J* = 4Hz), 10.24 (s, 1H, N*H*) ppm; MS (EI, 70 eV): *m/z* 399 [M+2]^+^, 397 [M]^+^; Anal. Calcd for C_21_H_16_ClNO_3_S: C, 63.39; H, 4.05; N, 3.52%. Found: C, 63.22; H, 3.91; N, 3.37%.

#### *N*-Phenyl-2-(4-(3-(thiophen-2-yl)acryloyl)phenoxy)acetamide (9a)


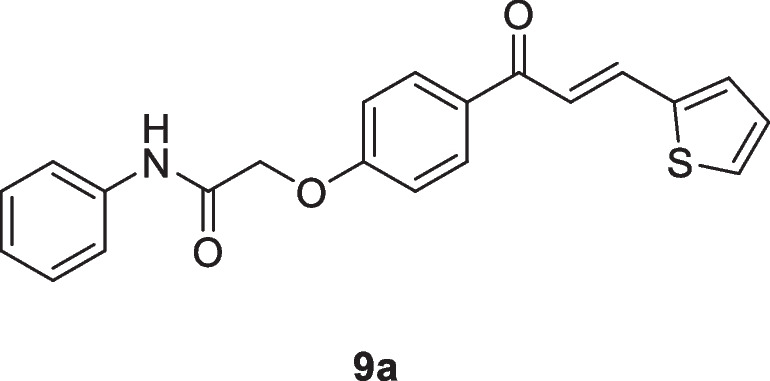


Yellow crystals (86%); mp 196-198 °C; IR (KBr): ν 3358 (NH), 1689 (C=O ketone), 1656 (C=O amide) cm^-1^; ^1^H NMR (400 MHz, DMSO-*d*_*6*_*)*: δ 4.86 (s, 2H, C*H*_2_), 7.08 – 7.21 (m, 4H, Ar-*H* + thiophene-H), 7.32-7.36 (m, 2H, Ar-*H*), 7.56 (d, 1H, + vinyl-*H, J* = 16Hz*),* 7.64 (d, 2H, Ar-H, *J* = 8Hz), 7.68 (d, 1H, thiophene-H*, J* = 4Hz), 7.77 (d, 1H, thiophene-H*, J* = 4Hz), 7.87 (d, 1H, + vinyl-*H, J* = 16Hz*)*, 8.12 (d, 2H, Ar-H, *J* = 8Hz), 10.18 (s, 1H, N*H*) ppm; ^13^C NMR (101 MHz, DMSO-*d*_*6*_*)*: δ 67.6 (-OCH_2_CO-), 115.2, 120.2, 120.8, 124.2, 129.1, 129.2, 130.6, 131.2, 131.4, 133.0, 136.5, 138.8, 140.3, 162.2 (ArC-O-CH_2_), 166.5 (-NHCO), 187.4 (-CO) ppm; MS (EI, 70 eV): *m/z* 363 [M]^+^; Anal. Calcd for C_21_H_17_NO_3_S: C, 69.40; H, 4.72; N, 3.85%. Found: C, 69.29; H, 4.65; N, 3.73%.

#### 2-(4-(3-(Thiophen-2-yl)acryloyl)phenoxy)-*N*-(p-tolyl)acetamide (9b)


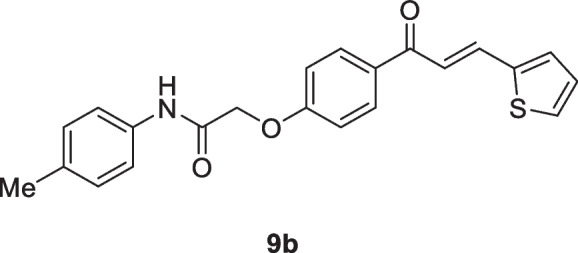


Yellow crystals (85%); mp 200-202 °C; IR (KBr): ν 3354 (NH), 1691 (C=O ketone), 1666 (C=O amide) cm^-1^; ^1^H NMR (400 MHz, DMSO-*d*_*6*_*)*: δ 2.27 (s, 3H, C*H*_3_), 4.83 (s, 2H, C*H*_2_), 7.13 – 7.22 (m, 5H, Ar-*H* + thiophene-H), 7.55 (d, 2H, Ar-H, *J* = 8Hz), 7.57 (d, 1H, vinyl-*H*, *J* = 20Hz), 7.70 (d, 1H, thiophene-H, *J* = 4Hz), 7.78 (d, 1H, thiophene-H, *J* = 4Hz), 7.90 (d, 1H, vinyl-*H*, *J* = 20Hz), 8.13 (d, 2H, Ar-H, *J* = 8Hz), 10.11 (s, 1H, N*H*) ppm; ^13^C NMR (101 MHz, DMSO-*d*_*6*_*)*: δ 20.9 (Me), 67.6 (-OCH_2_CO-), 115.2, 120.2, 120.8, 129.1, 129.6, 130.6, 131.2, 131.3, 133.0, 133.2, 136.3, 136.5, 140.3, 162.2 (ArC-O-CH_2_), 166.2 (-NHCO), 187.4 (-CO) ppm; MS (EI, 70 eV): *m/z* 377 [M]^+^; Anal. Calcd for C_22_H_19_NO_3_S: C, 70.01; H, 5.07; N, 3.71%. Found: C, 69.89; H, 4.95; N, 3.58%.

#### *N*-(4-Methoxyphenyl)-2-(4-(3-(thiophen-2-yl)acryloyl)phenoxy)acetamide (9c)


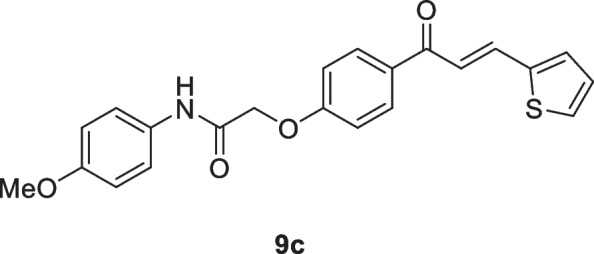


Green crystals (83%); mp 205-207 °C; IR (KBr): ν 3361 (NH), 1697 (C=O ketone), 1667 (C=O amide) cm^-1^; ^1^H NMR (400 MHz, DMSO-*d*_*6*_*)*: δ 3.74 (s, 3H, OC*H*_3_), 4.81 (s, 2H, C*H*_2_), 6.90 (d, 2H, Ar-H, *J* = 8Hz), 7.14 (d, 2H, Ar-H, *J* = 8Hz), 7.20 (m, 1H, thiophene-H), 7.54-7.60 (m, 3H, Ar-H + vinyl-*H*), 7.68 (d, 1H, thiophene-H, *J* = 4Hz), 7.77 (d, 1H, thiophene-H, *J* = 4Hz), 7.87 (d, 1H, vinyl-H, *J* = 16 Hz), 8.12 (d, 2H, Ar-H, *J* = 8Hz),10.03 (s, 1H, N*H*) ppm; ^13^C NMR (101 MHz, DMSO-*d*_*6*_*)*: δ 55.6 (OMe), 67.6 (-OCH_2_CO-), 114.3, 115.2, 120.8, 121.8, 129.1, 130.6, 131.2, 131.3, 131.8, 133.0, 136.5, 140.3, 156.1, 162.2 (ArC-O-CH_2_), 166.0 (-NHCO), 187.4 (-CO) ppm; MS (EI, 70 eV): *m/z* 393 [M]^+^; Anal. Calcd for C_22_H_19_NO_4_S: C, 67.16; H, 4.87; N, 3.56%. Found: C, 67.06; H, 4.80; N, 3.44%.

#### *N*-(4-Chlorophenyl)-2-(4-(3-(thiophen-2-yl)acryloyl)phenoxy)acetamide (9d)


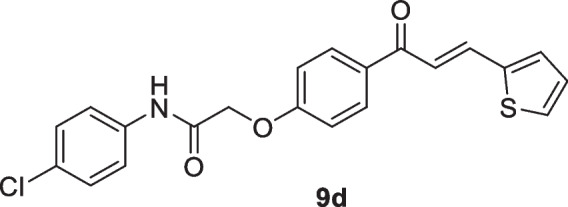


Yellow crystals (77%); mp 193-195 °C; IR (KBr): ν 3362 (NH), 1699 (C=O ketone), 1661 (C=O amide) cm^-1^; ^1^H NMR (400 MHz, DMSO-*d*_*6*_*)*: δ 4.86 (s, 2H, C*H*_2_), 7.14 (d, 2H, Ar-*H*, *J* = 8Hz), 7.19-7.21 (m, 1H, thiophene-H), 7.39 (d, 2H, Ar-H, *J* = 8Hz), 7.56 (d, 1H, vinyl-H, *J* = 16 Hz), 7.76-7.70 (m, 3H, Ar-H), 7.77 (d, 1H, thiophene-H, *J* = 8Hz), 7.87 (d, 1H, vinyl-H, *J* = 16 Hz), 8.12 (d, 2H, Ar-H, *J* = 8Hz),10.31 (s, 1H, N*H*) ppm; ^13^C NMR (101 MHz, DMSO-*d*_*6*_*)*: δ 67.5 (-OCH_2_CO-), 115.2, 120.8, 121.7, 127.9, 129.1, 130.6, 131.2, 131.4, 132.6, 133.0, 136.5, 137.8, 140.3, 162.1 (ArC-O-CH_2_), 166.7 (-NHCO), 187.3 (-CO) ppm; MS (EI, 70 eV): *m/z* 399 [M+2]^+^, 397 [M]^+^; Anal. Calcd for C_21_H_16_ClNO_3_S: C, 63.39; H, 4.05; N, 3.52%. Found: C, 63.25; H, 4.01; N, 3.41%.

### Cytotoxic Sulforhodamine-B (SRB) assay

The human laryngeal carcinoma (HEP2), colorectal carcinoma (HCT_116_), breast carcinoma (MCF7), Lung carcinoma (A549), liver carcinoma (HEPG2), and normal African Green monkey kidney cell line (VERO) were purchased from American Tissue Culture Collection (Rockville, MD, USA). The cells were treated for 48 h with a single dose (100 µg/mL) of all the tested chalcones. Then the IC_50_ was calculated for the most active compound **5c** against MCF7 and HEP2 cells using different concentrations (50, 25, 12.5, 6.25, 0.0 µg/mL). In brief, the cells were seeded in a 96-well microtiter plate at a concentration of 5×10^3^ cells/well in 100 µL fresh RPMI-1640 medium and left to attach to the plates for 24 h. Then, cells were incubated with 100 μL of different concentrations (50, 25, 12.5, 6.25, and 0.0 µg/mL) of **5c** in triplicate at 37 °C for 48 h. After that, the cells were fixed with 10 µL cold 100% Trichloroacetic acid (TCA) for 1h at 4 ºC. The wells were then washed 1 time with distilled water using (automatic Tecan washer, Germany) and stained for 30 min at room temperature with 50 µL 0.4% SRB dissolved in 1% acetic acid. The plates were air-dried and the dye was solubilized with 100 µl/well of 1M tris base (pH 10.5) for 5 min. The optical density (O.D.) of each well was measured spectrophotometrically at 570 nm with an ELISA microplate reader (Sunrise Tecan reader, Germany) with automatic shaking for 30 seconds before reading. The mean background absorbance was subtracted automatically and mean values for each drug concentration were calculated. The percentage of cell survival was calculated as follows: Survival fraction= O.D. (treated cells)/ O.D. (control cells) (Bhat et al. 2023).

### Real-time PCR

The expression level of the following six genes (*Ki-67, survivin, AKT1, IL-6, COX2,* and *IL-1B*) was examined using real-time polymerase chain reaction (qPCR) (Mohamed et al. 2023a). Total RNA was extracted from the treated and control samples using the RNeasy Mini Kit from Qiagen in Valencia (catalog#74104). cDNA synthesis was carried out using the High-capacity cDNA kit (Applied Biosystem, California, USA, Catalog #4368814) following the manufacturer's instructions. The qPCR was carried out following the manufacturer's instructions using the Promega GoTaq qPCR master mix (Madison, USA, Catalog# A6001). 25 µl of master mix, 0.25 µl of Carboxy-X-Rhodamine (CXR) reference dye, 1 µl of forward and reverse primers, 1µl of cDNA, and 50 µl of total volume was completed. Table 8 shows the sequences of the primers used. All analyses were carried out in triplicate on a 7500 Fast Real-Time PCR System (Applied Biosystems, Foster City, CA, USA) using a protocol that included an initial denaturation step at 95 °C for 10 minutes, followed by 40 cycles of denaturation at 95 °C for 15 seconds, and annealing at 62 °C for 1 minute. The cycle threshold (Ct) was determined automatically. Analysis of data was performed by using the ∆∆Ct method (Livak and Schmittgen 2001). Values were presented as relative expression levels and normalized to GAPDH.

**Table 8:** Forward and reverse primers of the studied genes

Gene	Forward	Reverse
GAPDH	**5-TGAAGGTCGGAGTCAACGGATTT-3**	**5-GCCATGGAATTTGCCATGGGTGG-3**
Survivin	**5- ACTTGGCCCAGTGGGTTTT-3**	**5- TGTTCCTCTATGGGGTCGTCA-3**
KI-67	**5- CAGGAAATCCTCAGCAACAAAA-3**	**5- TGGCTTAGTGGCAGAGTCAG-3**
AKT1	**5- GGAGGTTTTTGGGCTTGCG-3**	**5- GTCCATGGTGTTCCTACCCA-3**
COX2	**5-CCCTTCCTTCGAAATGCAAT-3**	**5- CATTTGAATCAGGAAGCTGC-3**
IL-6	**5-GAGACTTGCCTGGTGAAAAT-3**	**5-CAGGGGTGGTTATTGCATCT-3**
IL-1β	**5-GGACAAGCTGAGGAAGATGGC-3**	**5-TTTTTTGCTGTGAGTCCCGG-3**

### ELISA

ELISA colorimetric assay was used to determine the concentration of the following proteins (BAX, BCL2, P53, Caspase8, Caspase9, MMP2, MMP9, GSH, MDA and IL-6) using the following colorimetric detection kits Cloud-Clone Corp, USA, North America catalog # (SEB343Mu, SEA778Ra, SEH009Hu, MBS452285, MBS2533895, MBS670150, MBS175780, CEA294Ge, CEA597Ge and SEA079Ra, respectively). In brief, the instructions were described in the above kits as followed (Mohamed et al. 2023c): 100 μL of standard, blank, and samples were added into the appropriate wells, covered with the plate sealer, and incubated for 1 hour at 37°C. After removing the liquid from each well, 100 μL of the prepared reagent A working solution was added to each well, and incubated for 1 hour at 37 °C. The solution was aspirated and washed three times with 350 μL of 1X Wash Solution to each well using a squirt bottle, and multi-channel pipette. Then, 100 μL of detection reagent B working solution was added to each well, and incubated for 30 minutes at 37 °C. 90 μL of substrate solution was added to each well, covered with a new plate sealer, and incubated for 10 - 20 minutes at 37 °C in the dark. Finally, 50 μL of stop solution was added to each well. The liquid turned yellow with the addition of a stop solution. The microplate reader was run and conducted the measurement at 450 nm. A standard curve was plotted to calculate the concentrations of the unknown samples and the controls.

### Flow cytometric assay of cell cycle

In DMEM-supplemented media, cells were plated in 12-well plates at a cell density of 6–8×10^5^ per well. After twenty-four hours, cells were cultured for an additional 48 hours with the IC_50_ of the target compound. The untreated cells were used as a negative control. After 48 h of incubation, the treated cells were centrifuged at 1,200 rpm and 4 °C for 10 min. After discarding the supernatant, the wells were then given a single phosphate-buffered saline (PBS) wash and then centrifuged for 10 min at 1,200 rpm. Trypsin/EDTA was used to collect the cells, and after one PBS wash, they were resuspended in 0.5 mL of 0.05% Triton X-100 for 10 min at room temperature. Each cell suspension was stained by being given 1 mL of 50 g/mL propidium iodide (PI) to be left at room temperature in the darkness for 20 minutes. The analysis of the cell cycle was done by a flow cytometer (Becton Dickenson (BD) FACSCalibur, USA) (Michalkova et al. 2022).

### Wound healing assay

MCF7 and HEP2 treated and untreated cells were detached from the tissue culture plate using 0.25% Trypsin-EDTA solution. Cells pellet was prepared in a 15 mL conical tube by centrifugation. The supernatant was aspirated, and the cells were re-suspended in culture media. The appropriate number of cells was platted in a 6-well plate for 100% confluence in 24 hours. In a sterile environment (typically a biosafety hood), a 200 μL pipette tip was used to press firmly against the top of the tissue culture plate and swiftly made a vertical wound down through the cell monolayer. Carefully, the media and cell debris were aspirated. Slowly, enough culture media was added against the good wall to cover the bottom of the well and avoid detaching additional cells. Following the generation and inspection of the wound an initial picture was taken. The tissue culture plate was placed in an incubator set at the appropriate temperature and CO_2_ concentration (typically 37 °C and 5% CO_2_). After 48 hours, the plate was removed from the incubator and placed under an inverted microscope to take a snapshot picture and check for wound closure (Justus et al. 2014).

### Molecular docking

The molecular simulation studies were achieved using the Molecular Operating Environment (MOE) version 2009.10 (Mohamed et al. 2023b). The target compound **5c** was drawn using the program builder interface and then subjected to local energy minimization using the included MOPAC. Afterward, the model was subjected to global energy minimization using systematic conformational search where RMS gradient and RMS distance were set at 0.01 kcal/mole and 0.1 A^o^, respectively. The X-ray crystallographic structure of P53 cancer mutant Y220C and Bcl2 proteins complexed with their co-crystallized ligands (PDB ID: 5O1H and 6QGG), respectively, were obtained from the protein database. The proteins were modified for molecular simulations as follows; firstly, the hydrogen atoms were added. Afterward, the unwanted co-ligands and water chains were deleted. Then, the MOE alpha site finder was used to determine the active site of selected proteins. Finally, after the step of self-docking of the modified protein with its co-crystallized ligand, it was then subjected to be docked with the target compound to detect the protein-ligand interactions at the active domain. The final result of the protein-ligand interactions was visualized in 2D and 3D forms through BIOVIA Discovery Studio V6.1.0.15350.

## Conclusion

Among the synthesized chalcones, **5c** and **9a** had the most promising cytotoxic activity against MCF7 and HEP2 cells. Compound **5c** was chosen for further molecular studies for its lower IC_50_ values than Chalcone **9a**. It was found that compound **5c** had anti-proliferative activity in MCF7 and HEP2 cells by downregulating the expression level of *AKT1*, *Ki-67,* and *survivin* genes. Also, it had an anti-inflammatory effect in MCF7 and HEP2 cells which was shown by decreasing the expression of *IL-1B*, *COX-2* genes, and IL-6 (at the gene and protein levels). In addition, the anti-invasive and anti-metastatic effects of chalcone **5c** were exerted in MCF7 and HEP2 cells *via* lowering the activity of MMP-2 and MMP-9 and these results were confirmed by wound healing assay. Chalcone **5c** decreased the concentration of MDA and enhanced the activity of GSH demonstrating the anti-oxidant activity in MCF7 and HEP2 cells. Chalcone **5c** triggered intrinsic and extrinsic pathways of apoptosis in MCF7 and HEP2 cells by lowering the concentration of Bcl2 and increasing the concentration of BAX, P53, and caspases-8 and -9. Our molecular docking study against P53 mutant Y220C and Bcl2 supported our results in the ELISA studies. Chalcone **5c** caused cell cycle arrest at the G0-G1 phase in MCF7 cells and G2-M in HEP2 cells. So, the above-mentioned results predicted that chalcone **5c** might be used as a chemotherapeutic agent for the treatment of breast and laryngeal cancer.

## Supplementary Information

Below is the link to the electronic supplementary material.

Supplementary Material 1.

## Data Availability

No datasets were generated or analysed during the current study.
