# Supplementary material for: Synthesis, cytotoxicity, anti-inflammatory, anti-metastatic and anti-oxidant activities of novel chalcones incorporating 2-phenoxy-N-arylacetamide and thiophene moieties: induction of apoptosis in MCF7 and HEP2 cells
Source: Naunyn Schmiedebergs Arch Pharmacol. 2024 Jul 9;397(12):10091–107. doi: 10.1007/s00210-024-03255-9 (PMC11582173; doi:10.1007/s00210-024-03255-9)
Supplement: Supplementary file 1 — Supplementary Material 1. [file 210_2024_3255_MOESM1_ESM.docx]

**Synthesis, cytotoxicity, anti-inflammatory, anti-metastatic and antioxidant activities of Novel chalcones incorporating 2-phenoxy-*N*-arylacetamide and thiophene moieties: Induction of Apoptosis in MCF7 and HEP2 cells**

Nada S. Ibrahim,^1^ Hager Ahmed Sayed,^1^ Marwa Sharaky,^2^ Hadeer M. Diab,^3^ Ahmed H. M. Elwahy,^3^* Ismail A. Abdelhamid^3^*

^1^Department of Chemistry (Biochemistry Division), Faculty of Science, Cairo University, Giza 12613, Egypt

^2^Pharmacology unit, Department of Cancer Biology, National Cancer Institute, Cairo University, Cairo, Egypt

^3^Department of Chemistry, Faculty of Science, Cairo University, Giza 12613, Egypt

* e-mail: aelwahy@cu.edu.eg, [ismail_shafy@cu.edu.eg](mailto:ismail_shafy@cu.edu.eg); [ismail_shafy@yahoo.com](mailto:ismail_shafy@yahoo.com)

**Supplementary Data**

The ^1^H and ^13^C NMR of new compounds:

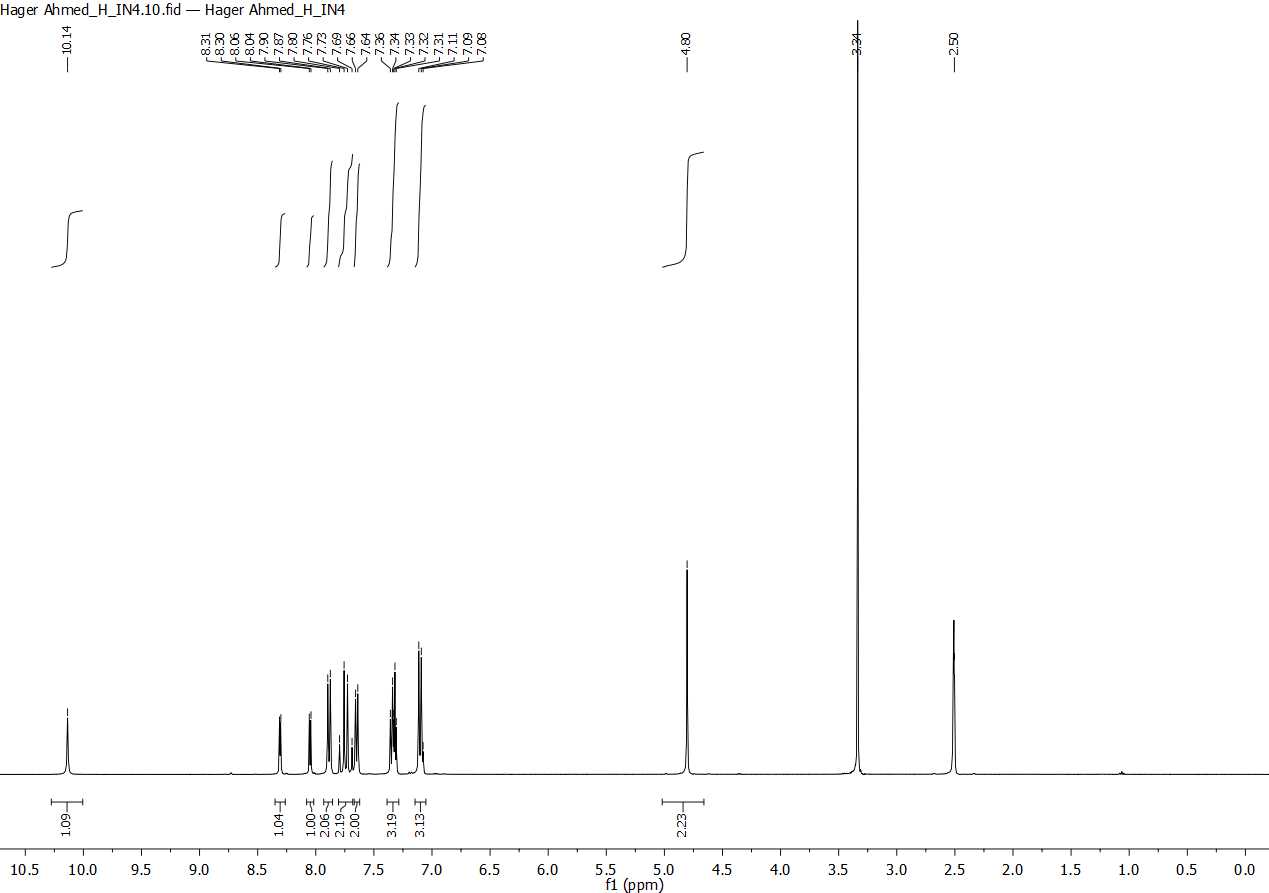


**Figure 1**. The ^1^H NMR spectrum of compound **5a**

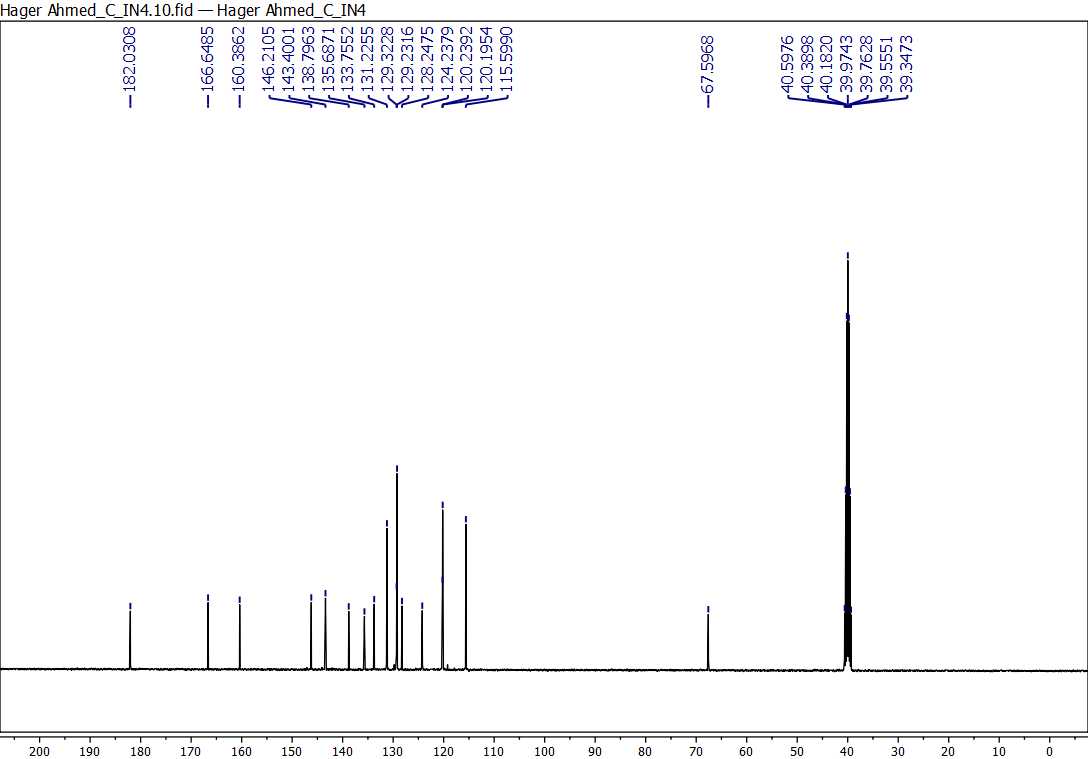


**Figure 2**. The ^13^C NMR spectrum of compound **5a**

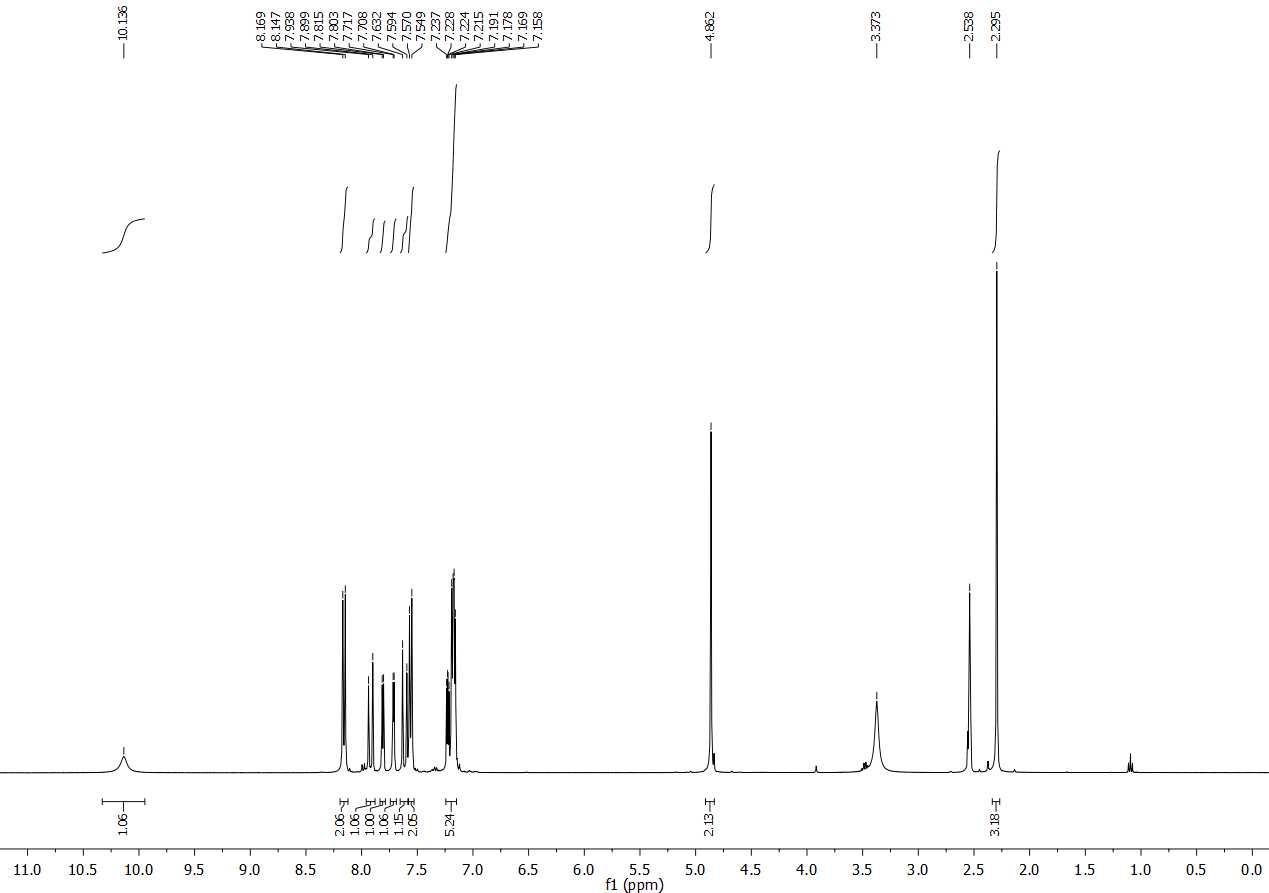


**Figure 3**. The ^1^H NMR spectrum of compound **5b**

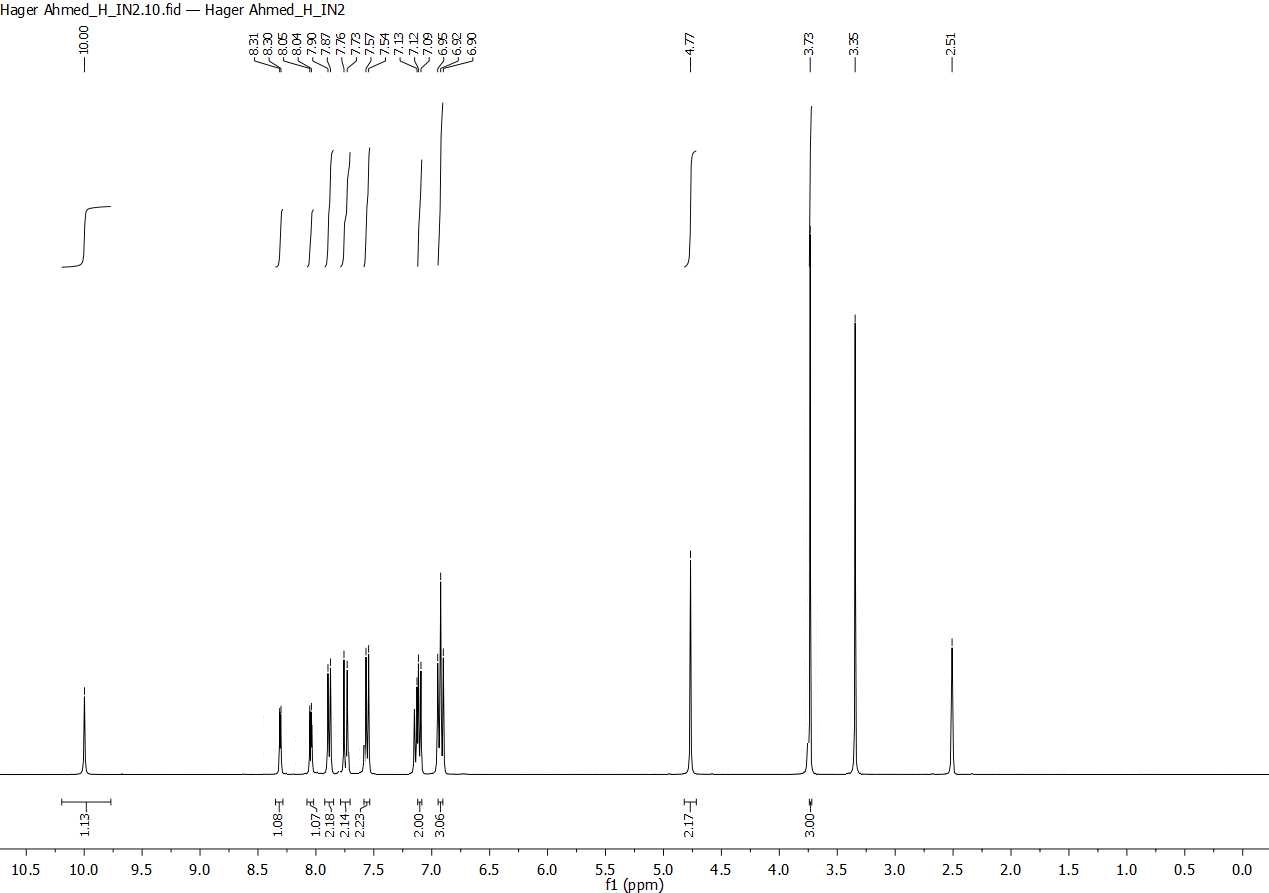


**Figure 4**. The ^1^H NMR spectrum of compound **5c**

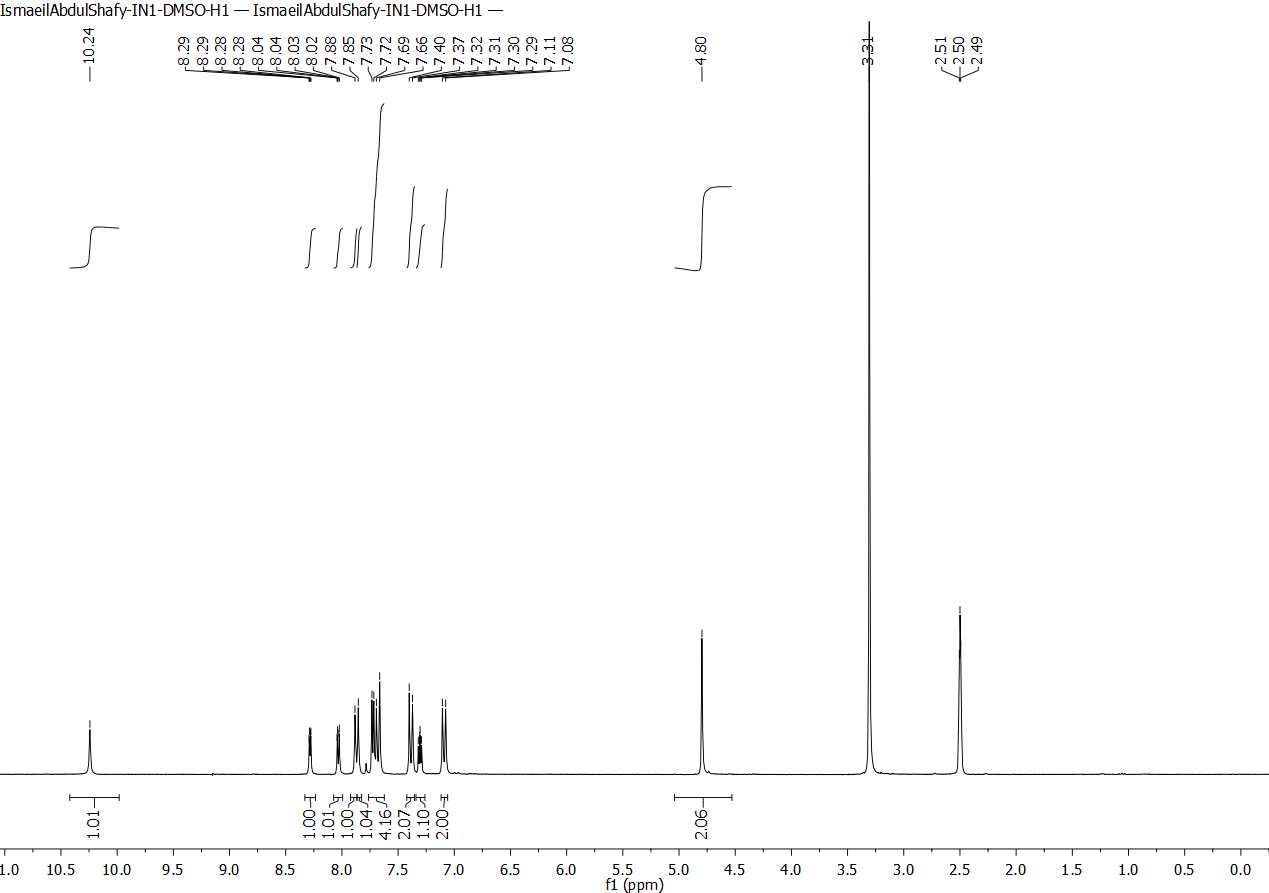


**Figure 5**. The ^1^H NMR spectrum of compound **5d**

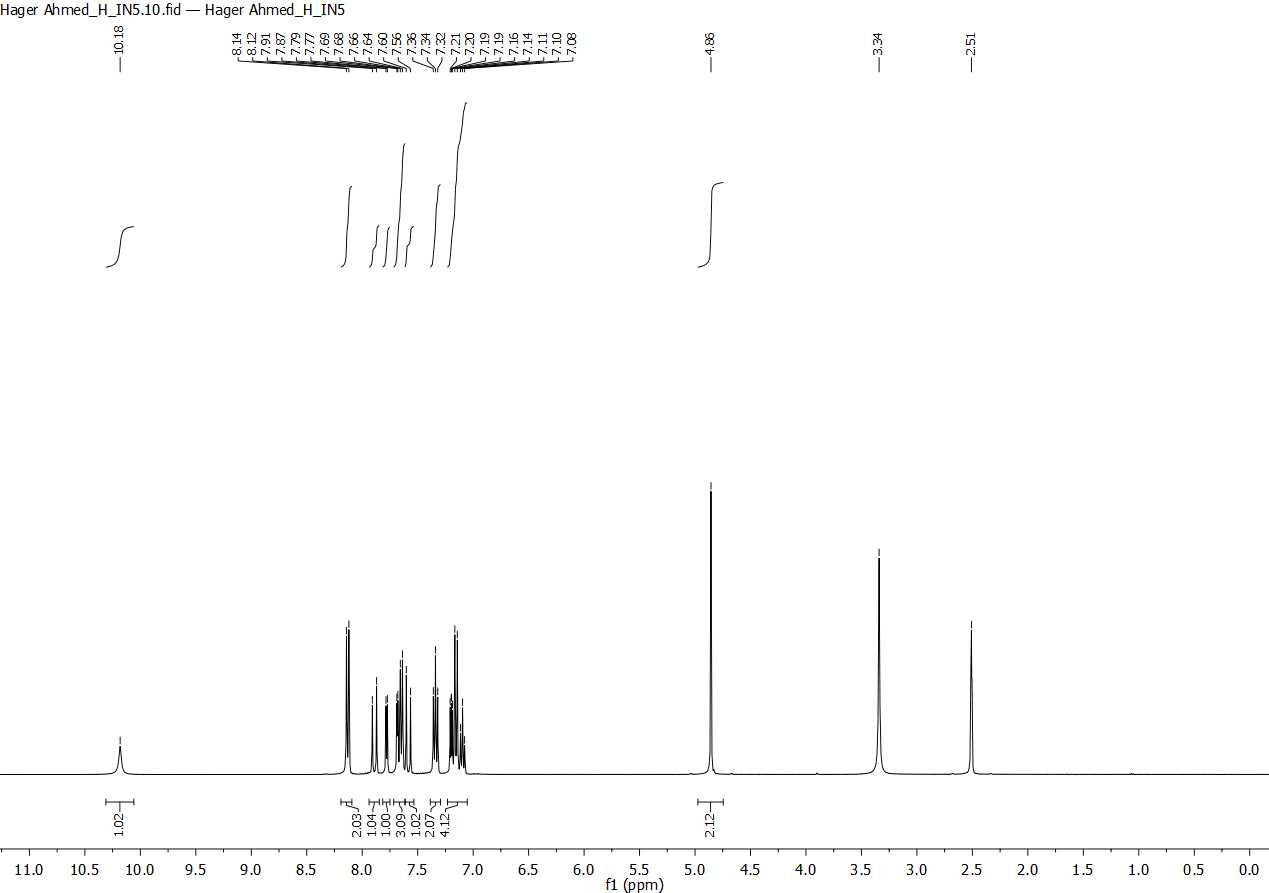


**Figure 6**. The ^1^H NMR spectrum of compound **9a**

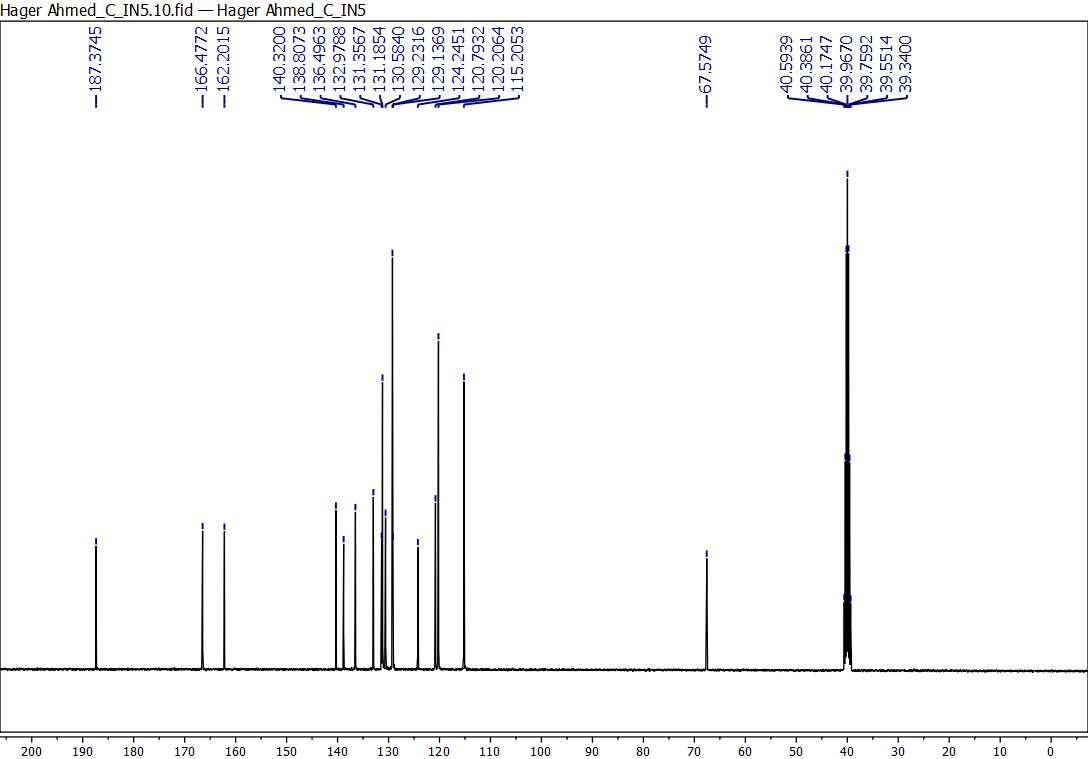


**Figure 7**. The ^13^C NMR spectrum of compound **9a**

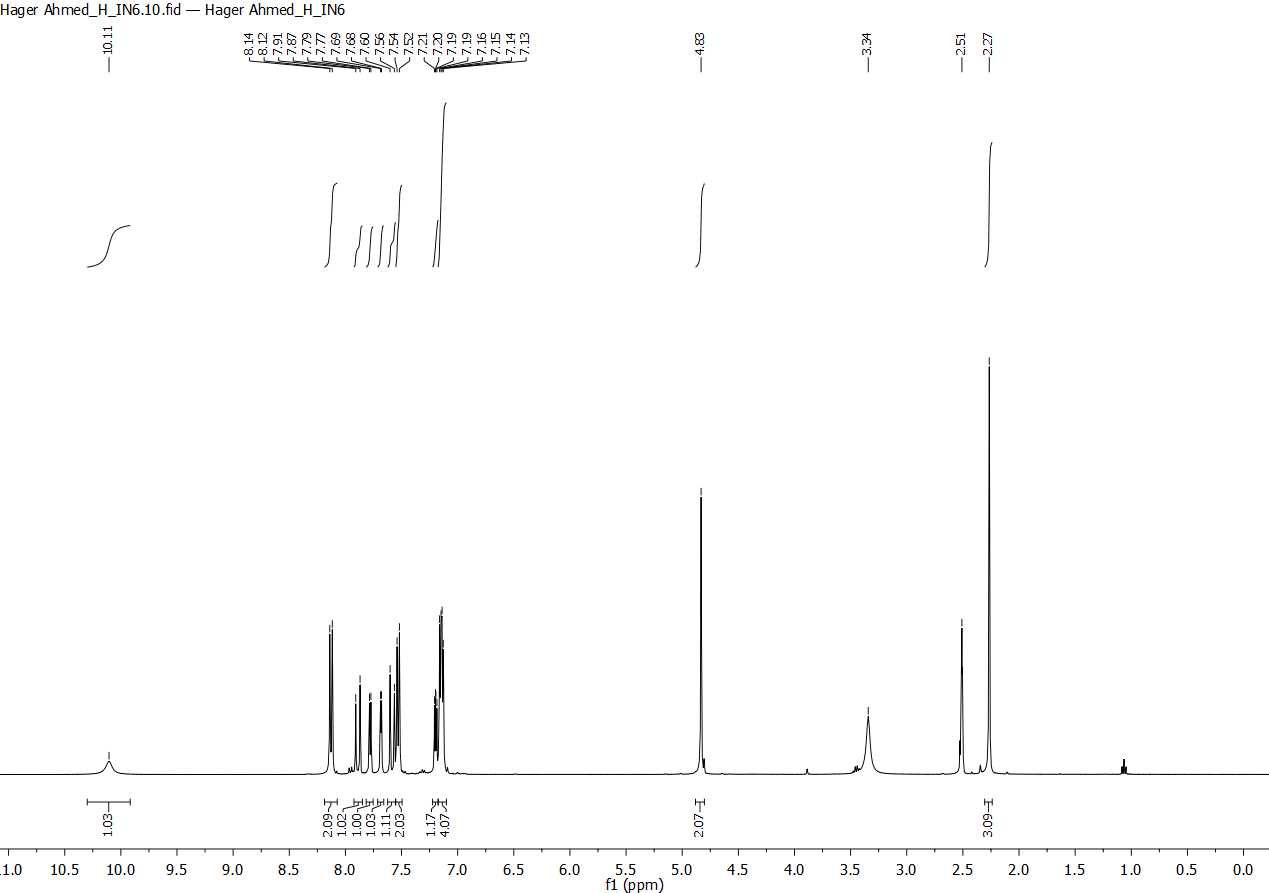


**Figure 8**. The ^1^H NMR spectrum of compound **9b**

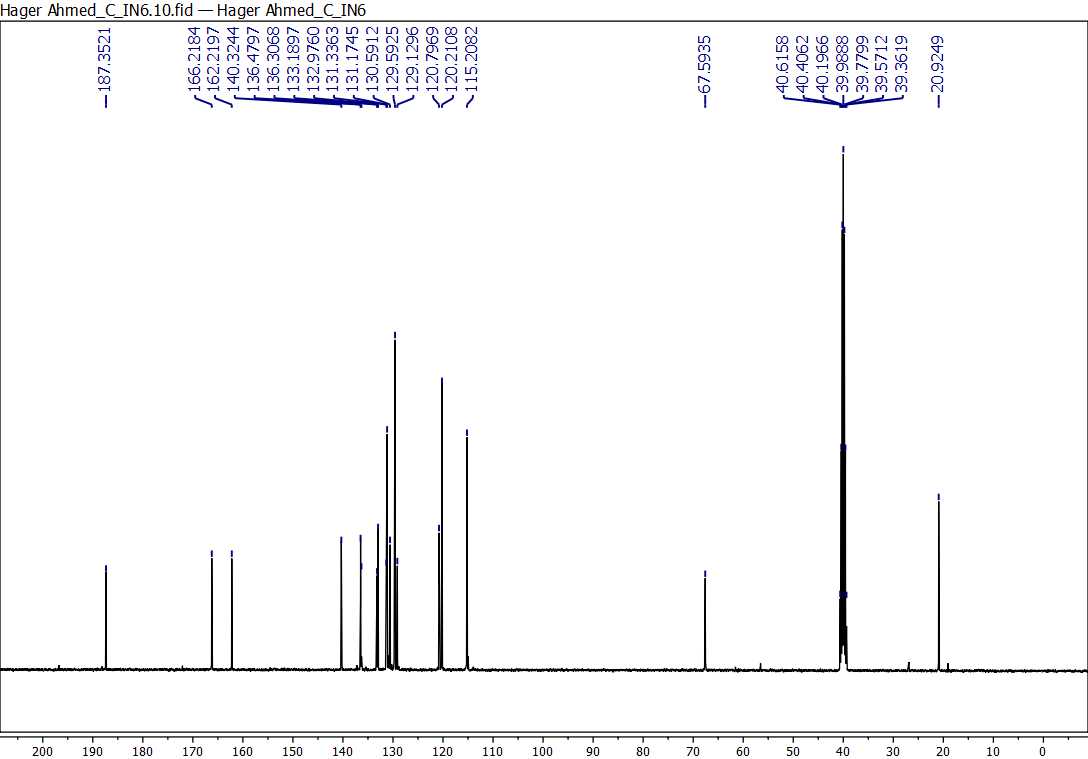


**Figure 9**. The ^13^C NMR spectrum of compound **9b**

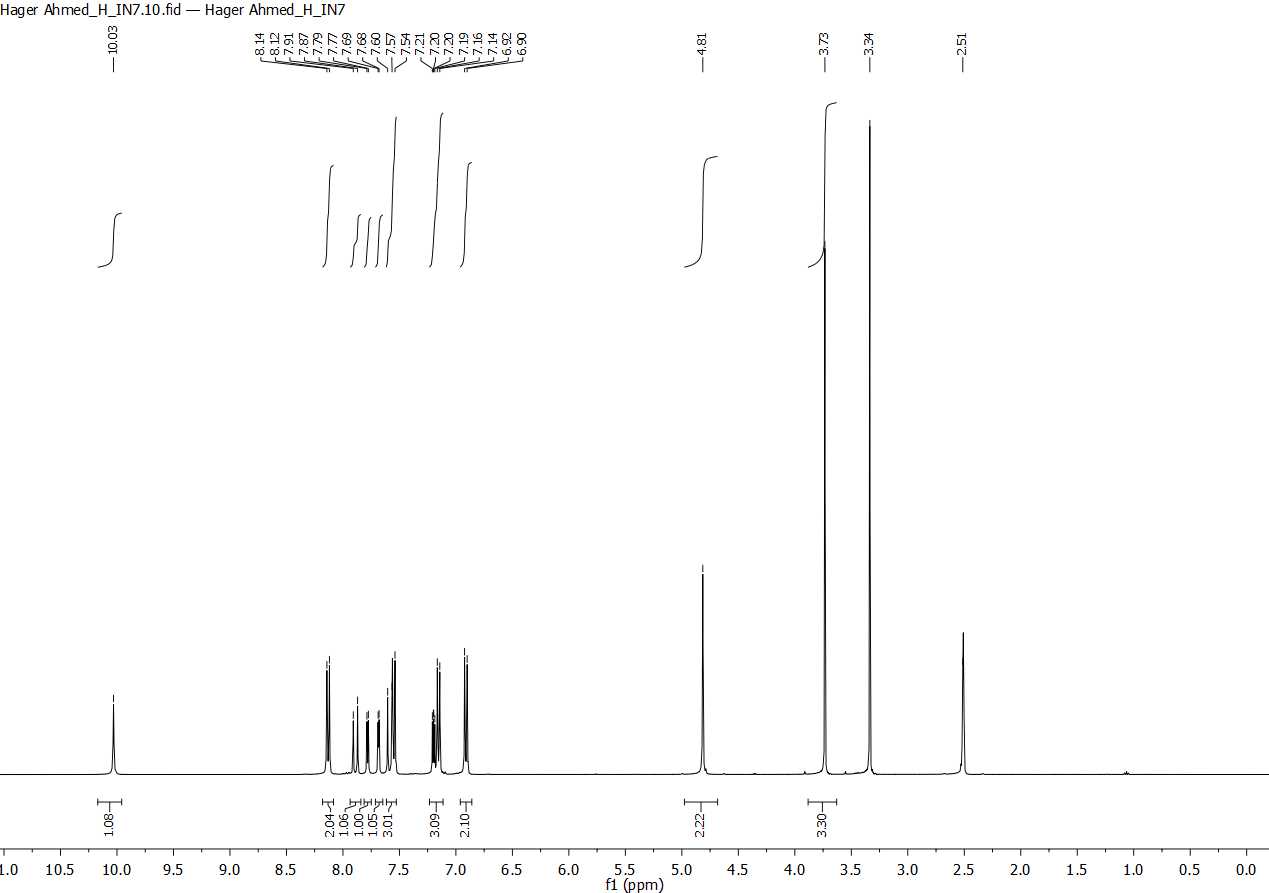


**Figure 10**. The ^1^H NMR spectrum of compound **9c**

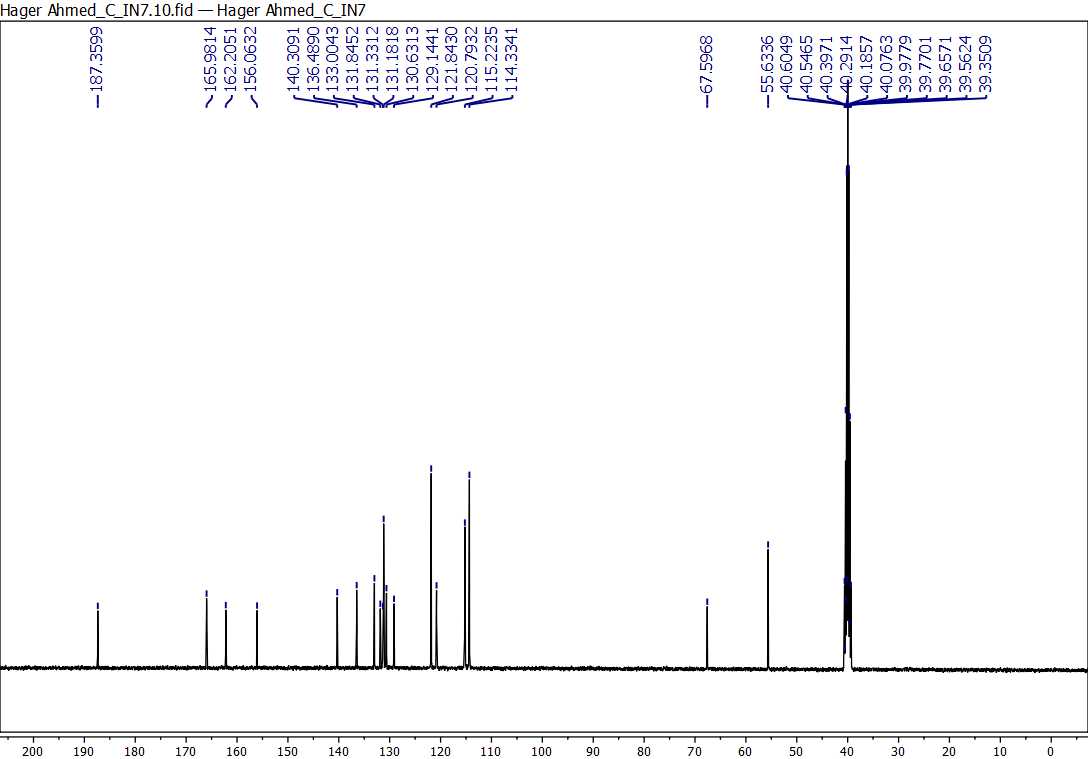


**Figure 11**. The ^13^C NMR spectrum of compound **9c**

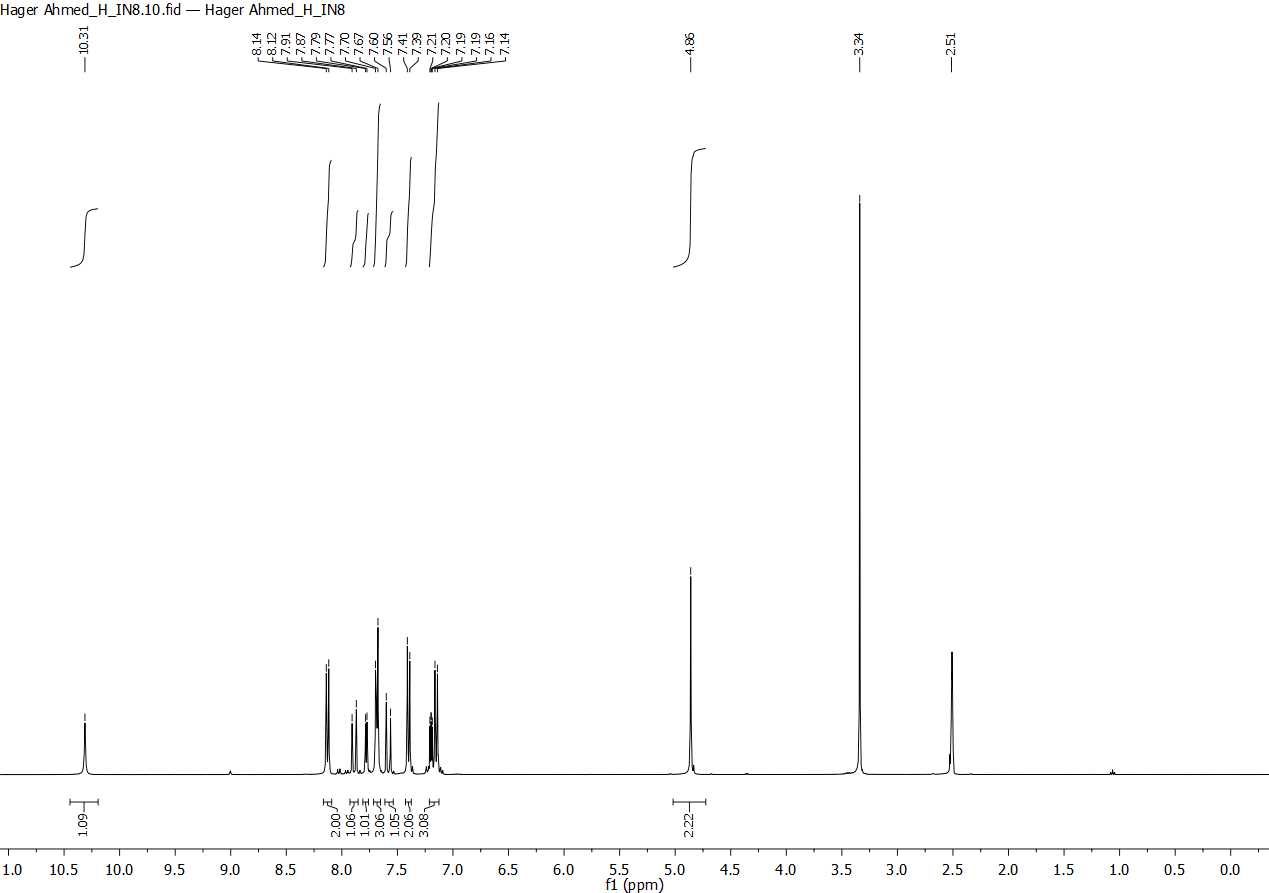


**Figure 12**. The ^1^H NMR spectrum of compound **9d**

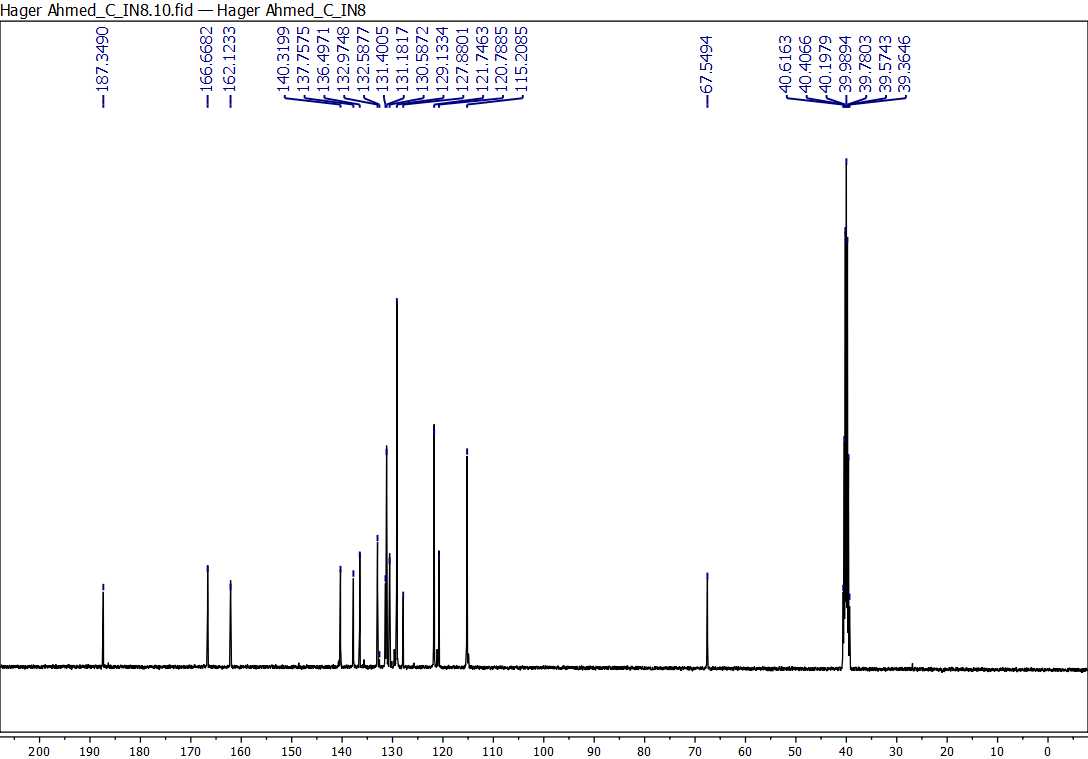


**Figure 13**. The ^13^C NMR spectrum of compound **9d**
